# Gut Microbiota Combined with Serum Metabolomics to Investigate the Hypoglycemic Effect of *Actinidia arguta* Leaves

**DOI:** 10.3390/nu15194115

**Published:** 2023-09-23

**Authors:** Yufei Hou, Lu Bai, Xin Wang, Shanshan Zhang, Shaojing Liu, Jiabing Hu, Jing Gao, Sen Guo, Chi-Tang Ho, Naisheng Bai

**Affiliations:** 1 College of Food Science and Technology, Northwest University, 229 Taibai North Road, Xi’an 710069, China; houyufei1988@126.com (Y.H.); guosen0828@163.com (S.G.); 2 Instrument Analysis Center, Xi’an Jiaotong University, 28 Xianning West Road, Xi’an 710048, China; 3 Department of Pharmaceutical Engineering, College of Chemical Engineering, Northwest University, 229 Taibai North Road, Xi’an 710069, China; 4 College of Pharmacy, Xi’an Medical University, 1 Xinwang Road, Xi’an 710021, China; 5 Department of Food Science, Rutgers University, 65 Dudley Road, New Brunswick, NJ 08901, USA

**Keywords:** *Actinidia arguta* leaves, type 2 diabetes mellitus, hypoglycemic effect, gut microbiota, serum metabonomics

## Abstract

*Actinidia arguta* leaves (AAL) are an excellent source of bioactive components for the food industry and possess many functional properties. However, the hypoglycemic effect and mechanism of AAL remain unclear. The aim of this work was to investigate the potential hypoglycemic effect of AAL and explore its possible mechanism using 16S rRNA sequencing and serum metabolomics in diabetic mice induced by high-fat feeding in combination with streptozotocin injection. A total of 25 flavonoids from AAL were isolated and characterized, and the contents of the extract from the AAL ranged from 0.14 mg/g DW to 8.97 mg/g DW. The compound quercetin (2) had the highest content of 8.97 ± 0.09 mg/g DW, and the compound kaempferol-3-*O*-(2′-*O*-D-glucopyl)-β-D-rutinoside (12) had the lowest content of 0.14 ± 0.01 mg/g DW. In vivo experimental studies showed that AAL reduced blood glucose and cholesterol levels, improved insulin sensitivity, and ameliorated oxidative stress and liver and kidney pathological damage. In addition, gut microbiota analysis found that AAL significantly reduced the F/B ratio, enriched the beneficial bacteria Bacteroides and Bifidobacterium, and inhibited the harmful bacteria Lactobacillus and Desulfovibrio, thereby playing an active role in intestinal imbalance. In addition, metabolomics analysis showed that AAL could improve amino acid metabolism and arachidonic acid metabolism, thereby exerting a hypoglycemic effect. This study confirmed that AAL can alleviate type 2 diabetes mellitus (T2DM) by regulating intestinal flora and interfering with related metabolic pathways, providing a scientific basis for its use as a dietary supplement and for further exploration of the mechanism of AAL against T2DM.

## 1. Introduction

Type 2 diabetes mellitus (T2DM) is characterized by hyperglycemia, hyperlipidemia, and relatively insufficient insulin, which seriously threatens human life and health [1,2]. Intestinal microbiota disorders and insulin resistance (IR) are linked to the pathogenesis of T2DM [3]. The clinical treatment of diabetes consists mainly of oral hypoglycemic drugs, including sulfonylureas, biguanides, α-glycosidase inhibitors, glucagon inhibitors, and insulin sensitizers, but they are usually accompanied by some side effects and are expensive [4,5]. Flavonoids are one of the most important phenolic chemicals in the plant kingdom. They have a range of biological activities, including hypoglycemic, hypolipidemic, antioxidant, and anti-inflammatory effects by reducing oxidation and inflammation, regulating glucose and lipid metabolism, and improving insulin resistance [6,7]. Therefore, exploring and discovering natural hypoglycemic and metabolic balance extracts rich in flavonoids with fewer side effects for preventing and treating diabetes is of great significance.

Increasing evidence supports that intestinal flora are directly correlated with the prevalence and development of T2DM [3,8,9,10,11]. The changes in the composition and function of intestinal flora are strongly associated with diabetic symptoms, such as hyperglycemia and IR [12]. Gut microbiota and its related metabolites are essential to the pathophysiological processes of T2DM, such as blood glucose metabolism, IR, and chronic inflammation [13]. Therefore, regulating the gut microbiota is essential to preventing and managing T2DM and related metabolic disorders.

Metabolomics is an emerging omics research technology following genomics, transcriptomics, and proteomics, which has been widely used to monitor the metabolites that have changed in organisms in real-time [14,15,16]. Metabolomics is characterized by high throughput and high sensitivity. Through the systematic study of metabolites in biological samples, the pathways linked to the illness process are found and the medication mechanism is elucidated to effectively reflect the metabolism in the body under specific conditions [17]. Chromatography–mass spectrometry (GC–MS, LC–MS, and CE–MS) and nuclear magnetic resonance technology are two frequently employed research methods in metabolomics [5,15,16,17,18,19]. Thus, exploring the hypoglycemic mechanism of natural extracts based on the combination of gut microbiota and metabolomics would have an important advantage.

The leaves of *Actinidia arguta* (AAL), as the by-product during the fruit ripening process, are discarded as crop waste, resulting in the waste of biological resources [20]. However, in China and Korea, AAL is used as a traditional edible material and as an exceptional source of value-added chemical substances for the food industry [20,21,22,23]. AAL has antioxidant, anti-inflammatory, anti-allergic, and antidiabetic effects [23,24,25].

Previously, the hypoglycemic effect of *A. arguta* leaves was investigated in vitro, but is still unclear in vivo [21,26]. Therefore, in this work, a T2DM model was created of a high-fat diet (HFD) and streptozotocin (STZ) injection to explore the antidiabetic effect of AAL in mice. Additionally, combining gut microbiota and metabolomics to elucidate the mechanism of the hypoglycemic effect of AAL.

## 2. Materials and Methods

### 2.1. Materials

The *Actinidia arguta* leaves (AAL) were gathered from the Xi’an Botanical Garden (Xi’an, Shaanxi, China), and authenticated by Prof. Naisheng Bai from the College of Food Science and Technology, Northwest University, Xi’an, China. Voucher specimens were deposited in our laboratory.

Forty SPF male C57BL/6 mice weighing 20 ± 2 g each were obtained from the Experimental Animal Center at Xi’an Jiaotong University (the production license number is SCXK (Shaan) 2018-001). The animal experiments were approved by the Northwest University Ethics Committee (AWC-20190202) and conducted in compliance with the guidelines on the Care and Use of Laboratory Animals at Northwest University.

Streptozotocin (STZ) was obtained from Sigma-Aldrich (St. Louis, MO, USA). Acetonitrile and formic acid were all UPLC grade, obtained from Fisher Chemical (Gaelic, Belgium) and Sigma-Aldrich, respectively. The deionized water was purified with the Milli-Q^®^ water purification system (Millipore, Burlington, MA, USA). The remaining materials were purchased from market resources and are now available.

### 2.2. Extraction, Characterization, and Quantification

The dried *A. arguta* leaves (15 kg) were added to 80% ethanol–water, and diafiltration was performed at room temperature three times for 24 h each time. Subsequently, the crude extract (1.15 kg) was dispersed with water and then extracted using petroleum ether and ethyl acetate. The water fraction (604 g) was obtained from water-layer raffinate after vacuum concentration.

The 580 g water fraction was used for column chromatography, and the residual part was vacuum freeze dried. The freeze-dried powder obtained was the sample used in this animal experiment, which was sealed at −80 °C. 

An integrated chromatographic separation approach using silica gel (100–200 mesh, 200–300 mesh, Qingdao Marine Chemical Inc., Qingdao, China), Sephadex LH-20 (Sigma Chemical Co., St Louis, MO, USA), MCI gel CHP20P (Mitsubishi Kasei Co., Tokyo, Japan), polyamide (60–100 mesh, Beijing Solarbio Science and Technology Co., Ltd., Beijing, China), and semi-preparative HPLC (Essentia Prep LC-16P, Shimadzu, Japan) was performed. The silica gel column was eluted with the MeOH-CH_2_Cl_2_ solvent system. The CHP-20P, LH-20, polyamide, and semi-preparative HPLC column chromatography were eluted with the MeOH-H_2_O solvent system. A total of 25 compounds were obtained: compounds **1** (17 mg), **2** (25 mg), **3** (20 mg), 4 (11.2 mg), **5** (13.2 mg), **6** (13.1 mg), **7** (11 mg), **8** (10.5 mg), **9** (15.9 mg), 10 (7.8 mg), 11 (10.9 mg), **12** (11.2 mg), 13 (10.4 mg), **14** (9.6 mg), **15** (6.9 mg), **16** (9.1 mg), **17** (7.7 mg), **18** (10.3 mg), **19** (18.3 mg), **20** (12.0 mg), **21** (13.1 mg), **22** (8.3 mg), **23** (9.5 mg), and **24** (11.2 mg). The detailed separation process is shown in Figure S1.

The compounds were evaluated using the Agilent 1260 Infinity HPLC system (Agilent Technologies Inc., Santa Clara, CA, USA). Chromatographic separations were performed at 30 °C on an Agilent HC-C18 column (250 mm × 4.6 mm, 5 µm). The mobile phase was composed of a gradient elution with 0.2% (*v*/*v*) phosphoric acid–water solution (A) and acetonitrile (B): 0 min, 5% B; 0–10 min, 5–25% B; 10–20 min, 25–50% B; 20–30 min, 50–75% B; 30–35 min, 75–95% B; 35–40 min, 95% B; 40–45 min, 95–5% B; and 45–50 min, 5% B. The flow rate was 1 mL/min, and the injection volume was 15 µL for all samples. The wavelength of the UV detector was set at 210 nm. The concentration of each component in the AAL was determined by HPLC analysis.

### 2.3. Animals and Administration

The HFD–STZ-induced T2DM mouse model was established, according to the previous method, in our lab [27]. Mice were raised in standardized conditions, where the temperature was 23 ± 2 °C, the relative humidity was 50 ± 10%, the lighting condition was 12 h light/12 h dark cycles, and there was free access to drinking and eating. After a week of adapted feeding, the mice were randomly grouped into four groups, with ten mice per group. One group continued to feed with an ordinary low-fat diet (LFD, 8% fat, 22% protein, and 70% carbohydrates), which was labeled as the control group, and the rest of the three groups were fed with a high-fat diet (HFD, 45% fat, 37% carbohydrates, and 18% protein; ReadyDietech, Shenzhen, China). After four weeks of feeding, the three HFD-fed groups received three consecutive days of intraperitoneal injections of 50 mg/kg body weight (BW) STZ, while the control group was given the same dosage of buffered citric acid–sodium citrate (Kemiou Chemical Reagent Co., Ltd., Tianjin, China). The diet was resumed two hours after the end of the injection and then stabilized for seven days. 

The HFD–STZ-induced mice with fasting blood glucose (FBG) levels ≥ 11.1 mmol/L were classified as T2DM mice [28]. And then, the T2DM mice were randomly grouped into the model group (Model), the AAL-treated group (AAL), and the metformin-treated group (Met). The mice in the control and model groups were gavaged with pure water, while the AAL-treated group mice were gavaged with 400 mg/kg BW of AAL, and the Met group mice were fed with 50 mg/kg BW of metformin. BW and FBG were recorded weekly throughout the treatment duration.

### 2.4. Sample Collection and Preparation

After the fifth week of feeding, the level of insulin (INS) and FBG were assayed, and the homeostasis model assessment: insulin resistance (HOMA-IR) was determined utilizing the following formula:

HOMA-IR = FBG (mmol/L) × INS (mIU/L)/22.5.

In addition, the oral glucose tolerance test (OGTT) was performed following 12 h of fasting. Following the intragastric administration of the glucose solution (1 g/kg BW), tail vein blood samples were taken at 0, 10, 20, 30, 60, 90, and 120 min.

The following day, 0.2 mL/20 g of 4% chloral hydrate was prepared for anesthetizing the mice, and then the mice were sacrificed to collect blood, liver, kidney, and feces samples.

Whole blood was obtained from the orbital, which was subsequently centrifuged for 15 min at 3000 rpm to gather the serum. At the same time, after the collection of liver and kidney tissues, each of them was divided into 2 pieces and washed 2–3 times with phosphate buffered saline (PBS); one was quick frozen in liquid nitrogen, and the other was kept in formalin for a histological assay. All of the obtained samples were kept at −80 °C following analysis. 

The preparation of the serum samples strictly followed the sample preparation standards proposed by the International Association of Metabolomics and were prepared according to the method described by Want et al. [29]. Specifically, pipet 100 μL of each serum sample, add 400 μL of acetonitrile–methanol (1:1, *v*/*v*) solution to the precipitate proteins, vortex for 30 s, sonicate in an ice bath for 10 min, place in a −20 °C refrigerator for 1 h, and then centrifuge at 12,000 rpm for 15 min at 4 °C. The supernatant was freeze dried, reconstituted with 100 μL of 2-chlorobenzalanine (4 ppm, purchased from Sigma-Aldrich, USA), an 80% methanol solution, 30 s of vortexing, 5 min of sonication on ice, and 15 min of 12,000 rpm centrifugation at 4 °C. The obtained upper solution layer was subsequently injected into a vial for UPLC-QTOF-MS^E^ analysis. To evaluate the system stability, a quality control (QC) sample was prepared by precisely pipetting 5 μL from each of the 24 serum samples. And the QC sample was randomly injected 5 times in the sample sequence.

### 2.5. Biochemical and Histological Assays

At the end of the experiment, the serum levels of triacylglycerols (TG), total cholesterol (TC), alanine aminotransferase (ALT), aspartate aminotransferase (AST), creatinine (Cre), blood urea nitrogen (BUN), and blood uric acid (BUA) were measured using commercial diagnostic kits, in accordance with the manufacturer’s standard procedures. The serum insulin level was determined by the double-antibody sandwich ELISA method. Part of the liver tissue was homogenized in normal saline, then centrifuged at 3000 rpm for 10 min at 4 °C, and the collection of supernatants was used to detect glutathione peroxidase (GSH-Px), malondialdehyde (MDA), and superoxide dismutase (SOD) using diagnostic kits. All the diagnostic kits were purchased from Nanjing Jiancheng Bioengineering Institute, Nanjing, China.

The liver and kidney tissues were embedded in paraffin following 24 h paraformaldehyde fixation. The serial slices (10 μm) were stained with hematoxylin and eosin (H&E). Finally, the histopathological changes in mice liver, kidney, hepatocyte degeneration, and cell necrosis were observed by using an optical microscope (7XB-PC, Shanghai Optical Instrument Factory, Shanghai, China).

### 2.6. UPLC-QTOF-MS^E^ Analysis Conditions

The UPLC-QTOF-MS^E^ analysis method refers to our previous research, with minor modifications [30]. The UPLC analysis was carried out using the Waters ACQUITY UPLC System (Milford, MA, USA). The ACQUITY UPLC BEH C18 column (100 mm × 2.1 mm, 1.7 μm) was applied for chromatography separation at 35 °C. The mobile phase was eluent A (0.1% formic acid in water, *v*/*v*) and eluent B (acetonitrile) at a 0.15 mL/min flow rate. The stepwise elution was optimized as follows: 0–2 min, 10% B; 2–3 min, 10–40% B; 3–16 min, 40–95% B; 16–18 min, 95% B; 18–20 min, 95–5% B; and 20–22 min, 5% B. The injection volume was set to 5 μL.

The Waters I-Class VION IMS QTOF mass spectrometry parameters were set as follows: the full scan range was *m*/*z* 50–2000, the capillary voltage floating was 2.00 kV (ESI+) or 2.50 kV (ESI−), the cone voltage was 40 V, the source temperature was 120 °C, the desolvation temperature was 600 °C, the cone gas flow was 50 L/h, and the desolvation gas flow was 800 L/h. In addition, the collision energy of the low energy function and the ramp collision energy of the high energy function were, respectively, set at 6.00 eV and 20–30 eV. The mass spectrometer was calibrated and locked by sodium formate and leucine enkephalin (*m*/*z* 556.2771 in positive ion mode; *m*/*z* 554.2615 in negative ion mode), respectively, to ensure mass accuracy and reproducibility.

### 2.7. Gut Microbiota Analysis of AAL Antidiabetic Effect

Firstly, the DNA was extracted from the colon contents of mice, and the DNA was quantified using the NanoDrop system, and the quality of the DNA extraction was assessed using 1.2% agarose gel electrophoresis. Then, the target fragment was amplified by PCR. Thirdly, magnetic beads were used to purify and recover the amplification products. Subsequently, fluorescence quantification of the PCR amplification-recovered products was performed. Finally, the Illumina MiSeq sequencing platform was utilized for high-throughput sequencing.

Sequence OTU clustering was performed using QIIME2 Dada2 analysis. According to the Greengenes database, species annotation was performed for each ASV characteristic sequence to obtain the taxonomic composition of the sample species. The alpha diversity level of the samples was estimated based on the distribution of ASV/OTU in different samples, and the suitable sequencing depth was represented by sparse curves. Principal coordinates analysis (PCoA) and non-metric multidimensional scaling analysis (NMDS) were applied to characterize the community differences between the samples, based on the Bray–Curtis distance. LEfSe (linear discriminant analysis effect size) analysis was performed to analyze species differences and to find robust marker species among the groups. According to the 16S rRNA gene sequencing results, we predict the bacterial community metabolic function of the samples and find out the differential pathways.

### 2.8. Metabolomics Analysis of AAL Antidiabetic Effect

The metabolomics method based on UPLC-Q/TOF-MS^E^ and multivariate statistical analysis were used to search for related small-molecule endogenous metabolites and related metabolic pathways in mice.

Firstly, the UNIFI scientific information system platform was used to collect the data, and the parameter settings refer to our previous research [30].

Secondly, the Progenesis QI software ver. 2.0 (Waters Corp., Milford, CT, USA) was used to preprocess the raw data collected from the UNIFI platform and identify the detected small-molecule metabolites. Data preprocessing mainly includes automatic alignment, peak extraction, and automatic deconvolution. For metabolite identification, there are now four levels of annotation: (1) identified substance with confidence (two orthogonal features supported by real chemical standard analysis performed in the same environment), (2) potentially identified compounds (based on one or two orthogonal qualities in a public database), (3) the potentially identified compound class, and (4) potentially unknown substance [31,32]. Therefore, the Progenesis QI search engine was used to query both publicly available databases (HMDB) and in-house databases. With the built-in Metascope for metabolite identification, compound IDs can be obtained according to up to five different criteria, including mass accuracy, retention time, fragment ion spectra, collision cross section (CCS), etc., which increase the confidence of metabolite identification, while minimizing false negative and false positive results [33]. For these orthogonal measures and allowing for a more balanced set of tolerance criteria, the parameters reported in our study are: a mass within 5 ppm, a retention time within 0.1 min, a CCS within 5%, and a relative mass error of performing theoretical fragmentation within 5 ppm. The MetaboAnalyst analysis platform [34] was used to perform multivariate statistical analysis, which involves principal component analysis (PCA) and orthogonal partial least-squares discriminant analysis (OPLS-DA), for screening the functional metabolites. Data normalization was carried out by median and Pareto scaling. Features with missing values that exceeded 50% were eliminated, and the remaining missing values were substituted with 1/5 of each variable’s minimum positive value. The interquartile range (IQR) filter was used to keep the variables close to a constant value throughout the experimental conditions. Two parameters, R2Y and Q2 of the permutation test, were used to assess the model’s validity and the reliability of the results [30]. In addition, we used VIP > 1, FC > 2 or FC < 0.5, and *p* < 0.05, as the criteria to screen potential differential markers. 

Finally, the identified potential biomarkers were imported into MetaboAnalyst 5.0 (http://www.Metabo-analyst.ca/ (accessed on 28 October 2022)), using HMDB (http://www.hmdb.ca/ (accessed on 28 October 2022)), KEGG (http://www. kegg.com/ (accessed on 28 October 2022)), and METLIN (http://metlin.scripps.edu/ (accessed on 28 October 2022)) databases for metabolic pathway analysis. The pathways with an impact > 0.10 in the MetaboAnalyst database are regarded as key potential biomarkers.

### 2.9. Statistical Analysis 

GraphPad Prism 8.0.2 software (GraphPad Software Inc., San Diego, CA, USA) was used to process the data. The Wilcoxon rank sum test, the Kruskal–Wallis H test, and the one-way ANOVA were employed to evaluate the differences. An LEfSe program was used to analyze the LEfSe. The correlations between the gut microbiota and metabolomic data were investigated using Spearman correlation analysis.

## 3. Results

### 3.1. Identification and Quantification

Twenty-five flavonoid compounds were isolated from AAL. These isolated compounds were identified by comparison with the NMR results reported in the literature (see the Supplementary Materials), and their chemical structures are displayed in Figure S2. They are isoquercitrin (**1**) [35], quercetin (**2**) [36], kaempferol (**3**) [36], catechin (**4**) [37], hyperoside (**5**) [38], kaempferol-3-rutinoside (**6**) [39], rutin (**7**) [40], quercitrin (**8**) [41], astragalin (**9**) [42], quercetin-3-*O*-rutinoside-7-*O*-glucoside (**10**) [43], quercetin-3-*O*-rutinoside-(1→2)-*O*-rhamnoside (**11**) [44], kaempferol-3-*O*-(2″-*O*-β-D-glucopyl)-β-D-rutinoside (**12**) [45], kaempferol-3-*O*-(2″,6″-α-L-dirhamnose)-β-D-glucoside (**13**) [44], kaempferol-3-sambubioside (**14**) [46], isorhamnetin-3-*O*-nehesperidine (**15**) [47], isorhamnetin-3-*O*-rutinoside (**16**) [47], isorhamnetin-3-*O*-β-D-glucoside (**17**) [47], 2″-*O*-galloylhyperin (**18**) [48], quercetin-4′-*O*-galactoside (**19**) [49], epigallocatechin (**20**) [50], taxifolin-7-*O*-rhamnoside (**21**) [51], quercetin-3-*O*-β-D-glucose-7-*O*-β-D-gentiobioside (**22**) [52], ampelopsin (**23**) [53], isorhamnetin (**24**) [54], and taxifolin (**25**) [55].

The quantification of the 25 compounds was established by HPLC (Figure S3), and the results are displayed in Table S1. The standard calibration curves of the analytes were established by plotting the relationship between the analyte concentration and the target peak area. The calibration curves of the 25 flavonoid compounds exhibited satisfactory linearity and correlation between the concentration and peak area over the linear range, with correlation coefficients of R^2^ ≥ 0.9991. The limit of detection (LOD) and limit of quantitation (LOQ) for all the analytes ranged from 0.02 to 2.34 μg/mL and from 0.60 to 7.12 μg/mL, respectively. The contents of the flavonoids in the extract from the *A. arguta* leaves ranged from 0.14 mg/g DW to 8.97 mg/g DW. The highest content in the compound was quercetin (**2**) with a content of 8.97 ± 0.09 mg/g DW, and the lowest content in the compound was kaempferol-3-*O*-(2′-*O*-D-glucosyl)-β-D-rutinoside (**12**) with a content of 0.14 ± 0.01 mg/g DW. The total flavonoid content in the extract was 41.03 mg/g DW.

### 3.2. Effects of AAL on Physiological and Biochemical Indices

**Figure 1:**
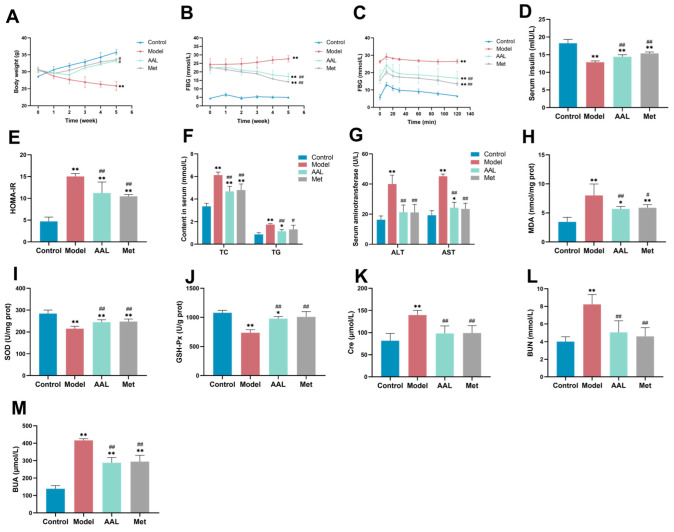
Effects of AAL treatment on the BW (**A**), FBG (**B**), OGTT (**C**), insulin (**D**), HOMA-IR (**E**), TC and TG (**F**), ALT and AST (**G**), MDA (**H**), SOD (**I**), GSH-Px (**J**), Cre (**K**), BUN (**L**), and BUA (**M**). The data are presented as mean ± SD (*n* = 6). In comparison with the control group (* *p* < 0.05, ** *p* < 0.01). In comparison with the model group (^#^ *p* < 0.05, ^##^ *p* < 0.01).

The BW of each group of mice was monitored weekly throughout the study (Figure 1A). In comparison to the control group, the BW of the diabetic model group was markedly reduced (*p* < 0.01), and after AAL and Met treatment for five weeks, the weight loss was markedly attenuated (*p* < 0.05). 

As illustrated in Figure 1B, the FBG levels in the mice in the model constantly grew after the development of the diabetes model (*p* < 0.01), while after five weeks of AAL and Met treatment, the FBG levels in the mice exhibited an obviously declining trend (*p* < 0.01). 

In the OGTT study, as shown in Figure 1C, the blood glucose levels in the mice in each group increased rapidly at 0–10 min, and after 10 min, the blood glucose levels in each group of mice first decreased sharply and then gradually tended to smooth out. Furthermore, the OGTT values were remarkably higher (*p* < 0.01) in the model group than the other groups. The INS study showed that the level of INS in the model group was considerably lower than that of the control group with *p* < 0.01, and after AAL and Met intervention, the level of INS was obviously higher with *p* < 0.01 (Figure 1D). As shown in Figure 1E, the HOMA-IR index for the model group was notably greater than that of the control group (*p* < 0.01), whereas the HOMA-IR indices in the AAL and Met groups were noticeably lower than those of the model group (*p* < 0.01).

The TC and TG levels were measured to evaluate the effects of AAL on the serum lipid profile (Figure 1F). In comparison with the control group, the levels of TG and TC in the model group were remarkably increased (*p* < 0.01), suggesting the presence of hyperlipidemia. After 5 weeks of treatment with AAL and Met, the levels of TG and TC in mice significantly declined compared to those in the model group (*p* < 0.01), indicating that AAL could alleviate dyslipidemia in diabetic mice.

The effects of AAL on liver function and oxidative stress in diabetic mice were investigated by detecting the levels of ALT and AST in serum and MDA, SOD, and GSH-Px in the liver. In comparison with the control group, the serum ALT, AST activity, and liver tissue MDA levels in the model group were significantly increasing (*p* < 0.01), while significantly decreasing in the AAL group (*p* < 0.01) and Met group (*p* < 0.05) (Figure 1G,H). In addition, as shown in Figure 1I,J, the SOD and GSH-Px levels in the model group were obviously lower than those of the control group, while those in the AAL and Met groups were greatly reversed. The results suggest that AAL could lessen liver injury and oxidative stress injury in diabetic mice. 

The renal function of diabetic mice was assessed using the serum levels of Cre, BUN, and BUA, and the results are displayed in Figure 1K–M. In comparison with the control group, the serum Cre, BUN, and BUA levels in the model group were markedly higher (*p* < 0.01). When compared with the model group, the contents of the serum Cre, BUN, and BUA in the AAL group were markedly lower (*p* < 0.01), demonstrating that AAL could improve renal function and alleviate renal injury in diabetic mice.

### 3.3. Histopathological Analysis

**Figure 2:**
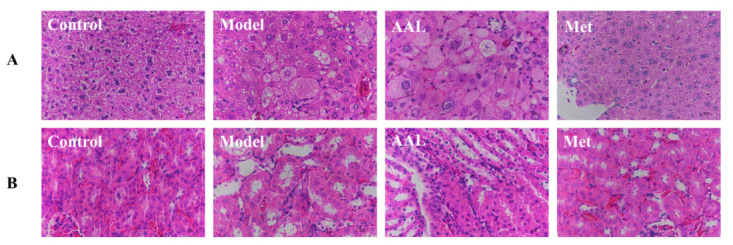
Effects of AAL treatment on tissue damage in the liver (**A**) and kidney (**B**) in diabetic mice.

To evaluate the effects of AAL on the liver and kidney histopathology in T2DM mice, the samples were stained with H&E to determine histopathological changes. The results (Figure 2A,B) show that the structure of the liver and kidney pathological sections from mice in the control group were clear, without obvious pathological damage. However, in the model group, the renal tissue of diabetic mice exhibited glomerular atrophy and basement membrane thickening, renal tubule edge blurring, swelling, congestion, and other histological injuries, and the liver of diabetic mice showed obvious fat vacuoles, disordered hepatocyte arrangement, inflammatory cell infiltration, and a liver cord disorder. After the intervention with AAL and Met, liver and kidney histological damage in diabetic mice was ameliorated.

### 3.4. Effects of AAL on Taxonomic Composition and Intestinal Microbial Diversity of Gut Microbiota

**Figure 3:**
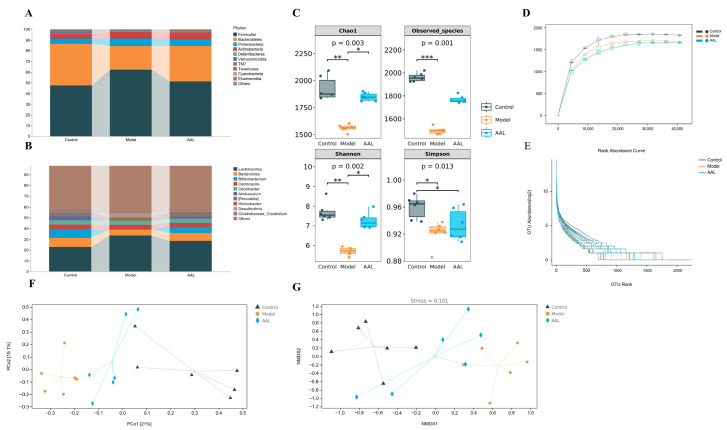
Effects of AAL treatment on regulation of gut microbiota community structure and diversity. Composition of intestinal microbial at the phylum level (**A**) and genus level (**B**) in the model, control, and AAL groups. Alpha-diversity index (**C**), including the Chao1 index, observed species index, Shannon index, and Simpson index (the Kruskal–Wallis test *p* value appears under the diversity index label, and the significant mark for Dunn’s post-hoc test is drawn by default; * *p* < 0.05, ** *p* < 0.01, *** *p* < 0.001). Alpha diversity analysis rarefaction curve (**D**) and rank abundance curve (**E**). The Bray–Curtis based PCoA (**F**) and NMDS analysis (**G**).

At the phylum level, Firmicutes, Bacteroidetes, Proteobacteria, and Actinobacteria were the dominant bacteria detected in all groups. As illustrated in Figure 3A, in comparison to the model group, the Firmicutes in the AAL intervention group remarkably dropped, while the Bacteroidetes increased obviously, and the Firmicutes to Bacteroidetes (F/B) ratio was significantly reduced (Figure S4). At the genus level, the Bacteroides, Bifidobacterium, Oscillospira, Allobaculum, (Prevotella), Odoribacter, and Clostridiaceae_Clostridium levels in the model group declined, while the Lactobacillus, Helicobacter, and Desulfovibrio levels increased (Figure 3B). After AAL administration, all of the above bacterial abundances were reversed, which suggests that AAL could regulate the community and structure of gut microbiota in T2DM mice.

For a comprehensive assessment of the alpha diversity of the microbial communities, the Chao1 and observed species indexes were determined to reflect the species richness, while the Shannon and Simpson indices were used to analyze the microbial biodiversity in the samples. The Chao1 index, observed species index, and Shannon and Simpson indices analysis indicated that the microbial community richness and diversity of the model group were obviously lower than those in the control group (Figure 3C). After AAL intervention, a rising trend in microbial community richness was observed, but with no marked difference between the AAL and the model group. As shown in Figure 3D, the rarefaction curve based on Chao1 was utilized to explore the changing trend in the alpha diversity with the leveling depth of the samples. The results showed a significant rise in the number of species detected in each group of samples with the deepening of the sequencing depth. However, with the increase in sequencing volume, the growth of the number of samples displayed by the two sparse curves slowed down significantly, while the Chao1 sparse curve entered a plateau when the sequencing volume increased to about 2000. In contrast, the rank abundance curve was able to visually reflect the amount of rare and high abundance ASV/OTU in the community. After AAL intervention, the more uniform distribution of the species and the higher species richness than that of the model group are shown in Figure 3E.

Additionally, beta diversity analysis was applied to compare the differences in the overall structure of the microbial communities in each sample. As illustrated in Figure 3F, the PCoA plot exhibited an obvious separation and spatial clustering, and the contributions from PCo1 and PCo2 were 21% and 15.1%, respectively. Interestingly, the intestinal flora of the mice in the AAL group was closer to the normal group along the PCo1 in comparison with the model group. Similarly, observations were obtained from the NMDS analysis, in which the intestinal flora of the normal and model groups were obviously separated, and the AAL treatment dramatically altered the intestinal microflora structure of the model group (Figure 3G).

### 3.5. Analyses of LEfSe Differential Marker Species and the PICRUSt2 Prediction Function

**Figure 4:**
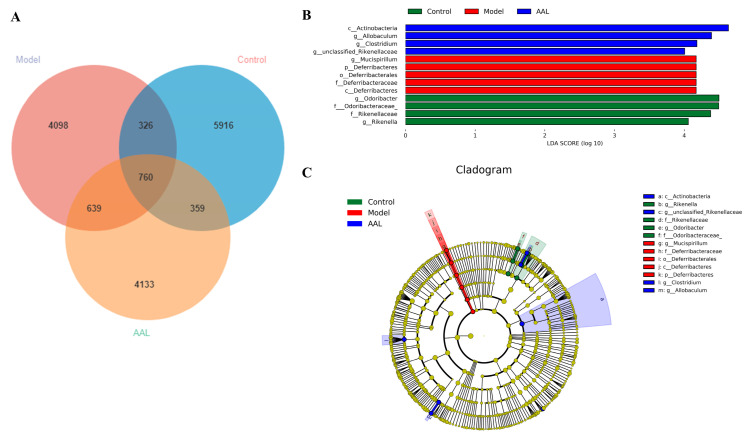

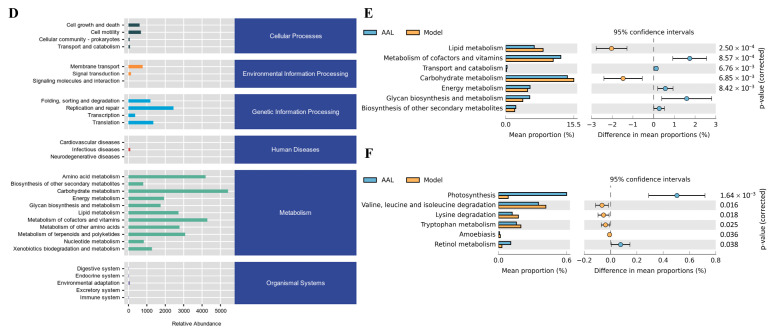
ASV/OTU expression in each group (**A**). The differential species LDA value distribution histogram (**B**) and the species taxonomy cladogram (**C**). Statistical diagrams of KEGG metabolic pathways (**D**). There was a significant differential in the KEGG metabolic pathways between the AAL-intervention group and T2DM mice at levels 2 (**E**) and 3 (**F**).

In Figure 4A, the overlapped ASV/OTU data in the Venn diagram displays 760 common OTUs for the three groups, as well as 5916, 4098, and 4133 unique microbes for the control, model, and AAL groups, respectively.

According to the above analysis, the gut flora in each group of mice varied in composition and structural aspects. Therefore, for finding robust biomarkers, the LEfSe analysis approach was applied to assess the variations between the three groups. The analysis results are illustrated by the taxonomic cladogram and LDA histogram (LDA score > 4), which show the taxonomic hierarchical distribution of the intestinal community species for each sample group and species significantly enriched within each group and their degree of importance, respectively. The LEfSe results (Figure 4B,C) show 13 robust differential biomarkers among the three groups at the taxonomic level above the genus. In the normal group, the biomarkers with an LDA score > 4 were Rikenellaceae, Rikenella, Odoribacteraceae, and Odoribacter. Likewise, Actinobacteria, Allobaculum, Clostridium, and unclassified Rikenbacteriaceae were remarkably enriched in the AAL group, and p_Deferribacteres, c_Deferribacteres, o_Deferribacterales, f_Deferribacteraceae, and Mucispirillum were screened in the model group.

The PICRUSt2 analysis was performed for functional prediction of the intestinal flora based on the KEGG database. As displayed in Figure 4D, we found that at the first classification level of KEGG, the gut microbiota was mainly involved in metabolism-related pathways, and 30 functional metabolic pathways were implicated in level 2 of the KEGG pathway. Among them, seven significantly different metabolic pathways were obtained between the model group and the AAL administration group at level 2 of the KEGG pathway, involving lipid metabolism, metabolism of cofactors and vitamins, transport and catabolism, carbohydrate metabolism, glycan biosynthesis and metabolism, and energy metabolism (Figure 4E). Further analysis revealed six significantly different pathways at level 3 of the KEGG pathway, including photosynthesis, valine, leucine and isoleucine degradation, lysine degradation, tryptophan metabolism, amoebiasis, and retinol metabolism (Figure 4F). The PICRUSt2 functional prediction analysis results suggested that AAL improved metabolic disorders in diabetic mice by ameliorating the intestinal flora imbalance. 

### 3.6. Serum Metabolomics Analysis

In this work, non-targeted metabolomics was applied to explore the serum metabolic profiles of T2DM mice after AAL treatment. As shown in the PCA score plot in positive and negative ion modes (Figure S5), each dot corresponds to a sample, and the QC samples were clustering tightly in the middle, which indicates that the stability and repeatability of the system were good. Meanwhile, the serum samples from the control, model and AAL groups were obviously divided into three different regions, and the AAL group was closer to the control group, indicating that the AAL intervention had a significant regulatory effect on the abnormal metabolic profile of T2DM mice.

**Figure 5:**
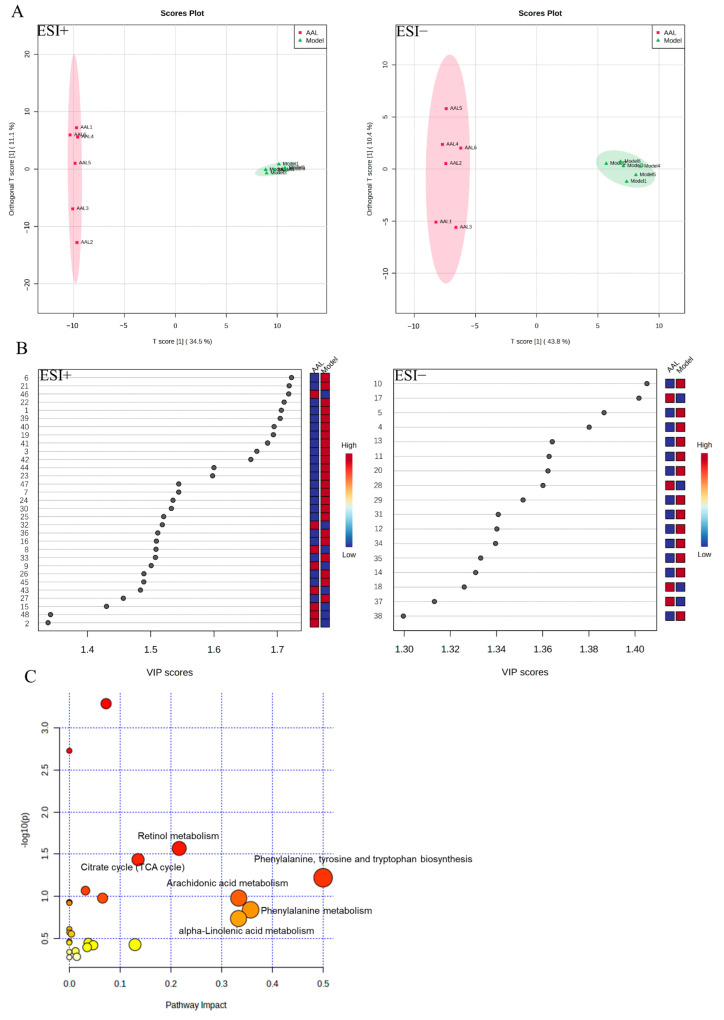
Serum metabolic profiling in positive and negative modes. The OPLS-DA score plots between the model group and the AAL group (**A**); the VIP score plots the model group and the AAL group (**B**); the metabolomics pathway analysis between the AAL group and the model group (**C**).

Further, an OPLS-DA model was established to distinguish the differences between the AAL and the model groups and obtain the differential metabolites of the AAL in the treatment of T2DM. As displayed in Figure 5A, a similar separation between the two groups was also observed. Simultaneously, a 100 permutation test for the OPLS-DA model was applied to assess the model’s reliability (Figure S6). In the positive ion mode, the interpretation rate of the OPLS-DA model of the serum samples was 0.997 (R2Y), and the prediction ability parameter Q2 was 0.954 (*p* < 0.01). In the negative ion mode, R2Y and Q2 were 0.987 and 0.955 (*p* < 0.01), respectively. The above results manifested that the established OPLS-DA model had a high interpretation rate and prediction rate, and that this OPLS-DA model was effective and reliable.

**Figure 6:**
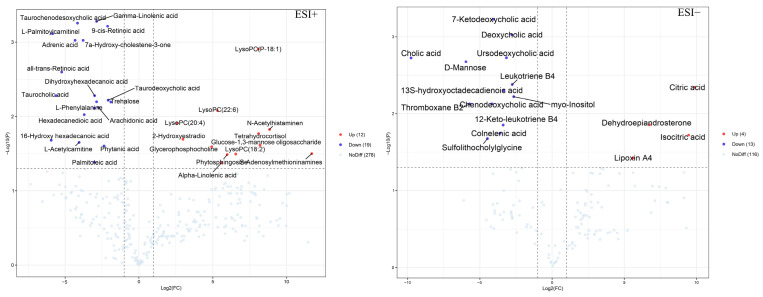
Volcano plots on the differential metabolites of the AAL vs. model.

A total of 48 differential metabolites that satisfied the conditions VIP > 1, FC > 2 or FC < 0.5, and *p* < 0.05 were screened and identified, the details of which are presented in Table S2. As shown in Figure 6, the volcano plot displays the relative changes in differential metabolites in two different groups. In comparison with the model group, 12 significant up-regulation metabolites and 19 significant down-regulation metabolites were shown in the AAL group in the positive ion mode, while 4 and 13 metabolites in the negative ion mode were notably up-regulated and down-regulated, respectively. Furthermore, the metabolic pathway analysis revealed that 48 different metabolites were primarily involved in six metabolic pathways with an impact value greater than 0.1, which were phenylalanine, tyrosine, and tryptophan biosynthesis; phenylalanine metabolism; arachidonic acid metabolism; alpha-linolenic acid metabolism; retinol metabolism; and citrate cycle (TCA cycle) (Figure 5C). Detailed information on the metabolic pathways of the differential metabolites is shown in Table S3.

### 3.7. Spearman Correlation Analysis 

**Figure 7:**
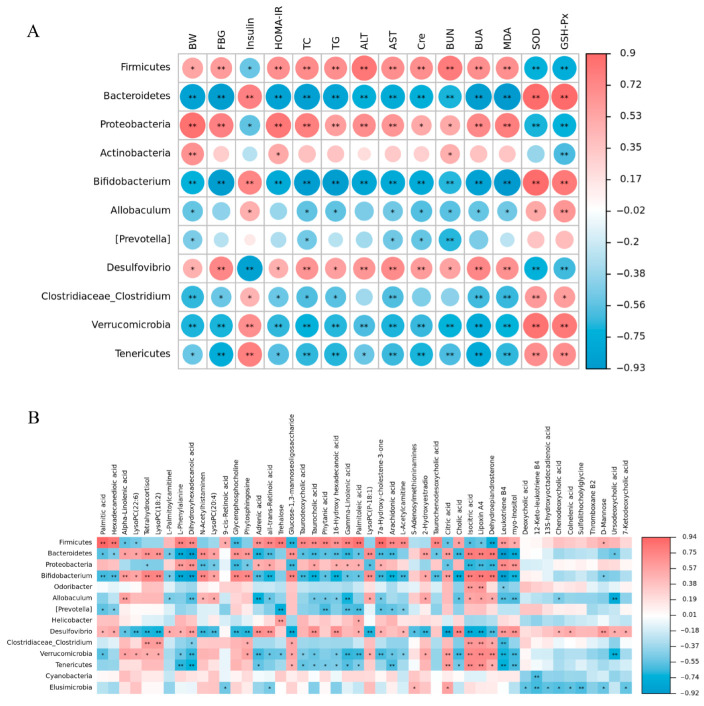
Correlation analysis of gut microbiota and T2DM risk factors in mice (**A**). Correlation analysis of gut microbiota and differential metabolites after AAL intervention (**B**). Blue represents a negative correlation, and red represents a positive correlation (* *p* < 0.05, ** *p* < 0.01).

The Spearman correlation analysis of the intestinal microbiota with factors related to T2DM and differential metabolites was performed using a free online gene cloud tool (https://www.genescloud.cn (accessed on 2 November 2022)), and the associated heatmap intuitively analyzed the correlation between them. According to the associated heatmap (Figure 7A), we found that the levels of T2DM-related parameters in the serum and liver had a positive or negative correlation with the abundance of gut bacteria. For instance, the factors BW, FBG, HOMA-IR, TC, TG, ALT, AST, Cre, BUN, and BUA in the serum were positively correlated with Firmicutes, Proteobacteria, and Desulfovibrio, and negatively correlated with Bacteroidetes, Bifidobacterium, Verrucomicrobia, Tenericutes, and Clostridiaceae_Clostridium. Similarly, the factors INS, SOD, and GSH-Px had significant positive correlations with Bifidobacterium, Verrucomicrobia, Tenericutes, and Clostridiaceae_Clostridium, while negatively correlated with Firmicutes, Proteobacteria, and Desulfovibrio. In addition, the BW factor had a strongly positive correlation with Actinobacteria, and BUN was negatively correlated with (Prevotella).

According to the correlation analysis heat map of the bacterial taxa and differential serum metabolites involved in AAL (Figure 7B), the relative abundance of Firmicutes was found to have a significantly positive correlation with the level of palmitic acid (r = 0.938, *p* < 0.01). Moreover, Bifidobacterium, a crucial gut probiotic, showed high positive correlations with lipoxin A4 (r = 0.814, *p* < 0.01) and dehydroepiandrosterone (r = 0.819, *p* < 0.01), which might be conducive to ameliorating the metabolic abnormalities in T2DM. 

## 4. Discussion

Our study found that AAL is rich in flavonoids, mainly flavonols, which is consistent with previous reports [20]. The content of quercetin was the highest (8.97 ± 0.09 mg/g DW), followed by kaempferol (6.89 ± 0.04 mg/g DW), and the content of quercetin and kaempferol were close. Wojdyło et al. found that the contents of quercetin derivatives (22.64 mg/100 g dw) and kaempferol derivatives were (18.40 mg/100 g dw) in *Actinidia arguta* fruits, which were lower than the content of the flavonols found in our study. Meanwhile, the two substances are often used as promising ingredients for food supplements and functional foods [56]. In addition, it has been reported that flavonols are a valuable phytochemical with antioxidant and anti-inflammatory activities that play an important role in the prevention and treatment of type 2 diabetes [57]. Therefore, based on the results in Table S1, it is speculated that the rich content of flavonols in AAL might be responsible for the hypoglycemic effect, which further clarifies that AAL could be used as an important source of active ingredients.

Typical symptoms of T2DM are BW loss, increased FBG, insulin resistance, and dyslipidemia [58]. In our study, these changes were significantly reversed after AAL intervention, which indicates that AAL could improve hyperglycemia and insulin resistance, enhance insulin sensitivity, and accelerate glucose metabolism. Moreover, AAL intervention could alleviate lipid metabolism abnormalities, lower TG and TC levels, and regulate blood lipids in T2DM mice. Persistent hyperglycemia caused by T2DM can lead to excessive intracellular reactive oxygen species (ROS) production through various mechanisms and insufficient clearance, thereby leading to oxidative stress, tissue damage, and oxidation [59]. In addition, GSH-Px and SOD protect cells from damage, while MDA is the most commonly used lipid marker of lipid peroxidation damage [5,60]. After AAL intervention, the MDA content decreased, and the SOD and GSH-Px levels increased, indicating that AAL could promote ROS scavenging and reduce tissue damage by enhancing antioxidant capacity. Diabetes may lead to abnormal liver and kidney function [61]. In this work, we discovered that AAL could mitigate liver and kidney damage in mice with T2DM. ALT and AST levels in serum are the most obvious biochemical indicators of the extent of liver cell injury, which were high in T2DM mice [62]. And after AAL treatment, serum ALT and AST were significantly reduced. Renal function injury can cause an increase in Cre, BUN, and BUA levels [63]. Remarkably, the treatment with AAL effectively decreased the levels of Cre, BUN, and BUA. Moreover, histopathological studies of the H&E-stained liver and kidney sections showed that AAL protects hepatocytes and kidneys against damage caused by T2DM. 

Alpha diversity is an indicator of species richness, diversity, and evenness in locally homogeneous habitats [64]. Previous studies have reported that diabetes affects the uniformity and diversity of gut microbiota [5,65], which is similar to our findings. At the same time, it is well known that the greater the Chao1 index and observed species index, the higher the community richness and the higher the Shannon index and Simpson index, the higher the community diversity [66,67,68]. Consistent with the above description, the alpha diversity indexes for the AAL group were greater than those for the model group, demonstrating a rising trend in the variety of microbial communities. The rarefaction curve is used to explain whether the sequencing data on the samples is reasonable, and the curve tends to be flat, exhibiting that the sequencing depth is reasonable [69,70]. Moreover, in the rank abundance curve, the length of the curve in the horizontal direction reflects the abundance of the species; the range of the curve on the horizontal axis increases with species richness. The uniformity of the species in the sample is reflected by the gentleness of the curve, which means the gentler the curve, the more uniformly the species are distributed [71]. Our findings coincide with the above descriptions. Beta diversity is the rate of species replacement or the variability in species composition along environmental gradients between various communities [72]. In this work, unconstrained sorting PCOA and NMDS were applied to visually display the differences in the microbial communities between the different groups. Among them, the NMDS plot usually gives the stress value of the model to determine whether the plot accurately reflects the true distribution of data orders [73]. The stress value in our NMDS analysis was 0.107, less than 0.2, and this suggests that the results from the NMDS analysis are reliable. 

Many studies have reported that the abundance of the two dominant bacterial species (Firmicutes and Bacteroidetes) was related to the obesity phenotype, and dietary supplements could have decreased the F/B ratio in HFD-fed T2DM mice [5,11]. In this study, Firmicutes presented a positive correlation with blood glucose, lipid levels, and oxidative stress levels, while Bacteroidetes were negatively correlated with them. Moreover, we observed a large rise in Firmicutes and an obvious drop in Bacteroidetes in T2DM mice, and the F/B ratio exhibited a striking reduction with the treatment with AAL, which agreed with the findings from several investigations [74,75].

Proteobacteria belong to gram-negative bacteria and contain a variety of pathogenic species, and intestinal flora imbalance and metabolic disorders are linked with increased Proteobacteria [76]. AAL supplementation down-regulated the level of Proteobacteria, but there was no significant change. According to the LEfSe analysis, Actinobacteria was identified as a significantly different species with a greater influence on the microbial community in the AAL group, which agreed with the previous study [5]. Verrucomicrobia is considered an effective intestinal bacteria for improving insulin sensitivity and glucose metabolism, and the decline of which might promote the development of T2DM [74]. According to the current study, Verrucomicrobia abundance was observed to be obviously declining in the T2DM group and rising markedly after AAL intervention, and was positively correlated with insulin and antioxidant levels. In addition, no obvious differences were found in the less frequent phyla of Tenericutes, TM7, and Deferribacteres with an abundance of <1%. Deferribacteres and its Mucispirillum genus are the representative species influencing the bacterial community in the model group.

The current research further described the variations in the genus-level abundance of bacterial taxa. Overall, the model group had a bacterial profile poor in Bacteroides, Bifidobacterium, Oscillospira, Allobaculum, (Prevotella), Odoribacter, and Clostridium, and rich in Lactobacillus, Helicobacter, and Desulfovibrio. Among them, Lactobacillus, Bacteroides, and Bifidobacterium were the most prevalent bacteria at the genus level. Various studies have reported that Lactobacillus was positively associated with metabolic disorders and the abundance of Lactobacillus was enriched in patients with T2DM and obesity [5,77,78]. In this work, the level of Lactobacillus in the T2DM group was greatly increased compared to that of the control group, relating to increased glucose levels in the intestine. Bacteroides play a beneficial or pathogenic role according to their position in the host, usually playing a beneficial role in the intestine [79]. Several previous studies have reported that the level of Bacteroides declined in T2DM compared to the control group, which is in line with our study [3,80,81]. Bifidobacteria is an important beneficial bacterium that can improve metabolic disorders in T2DM [5,80,82]. This study showed a significant increase in the relative abundance of Bifidobacteria in the AAL group and a positive correlation with insulin, SOD, and GSH-Px levels. Genera Oscillospira, Allobaculum, and Clostridium belong to the phylum Firmicutes, among which beneficial bacteria Oscillospira and Allobaculum are considered to have a close relationship with human health. The abundance of the two genera in the intestine of patients with obesity and obesity-related metabolic diseases is significantly reduced and negatively correlated with FBG, TG, and BUA [83,84,85]. Some studies have reported that a high abundance of Clostridium is related to obesity and metabolic disorders [86,87], while others have shown the opposite [88,89]. This study found that the levels of Oscillospira, Allobaculum, and Clostridium obviously declined in T2DM mice, and AAL intervention could significantly reverse the reduction of the three genera. In the correlation analysis, which intuitively showed that Allobaculum and Clostridium were negatively correlated with serum lipid levels, a significantly positive correlation with GSH-Px was also shown [85,90]. Further, LEfSe analysis verified that Allobaculum and Clostridium are key phenotypes of the intestinal flora in the AAL group. Odoribacter as part of a healthy, balanced gut microbiota belongs to the Bacteroidetes species. Based on previous studies, the reduced abundance of Odoribacter is associated with metabolic-related diseases, while a higher abundance of it in the intestine could improve insulin sensitivity [77,91]. In accordance with previous research, this study found that Odoribacter abundance was less prevalent in the model group than in the normal group, and AAL intervention slightly but not significantly increased its abundance. According to LEfSe analyses, Odoribacter and Odoribacteraceae were abundant in the normal group. Therefore, this might be the potential mechanism for AAL to alleviate diabetes by modulating the structure and diversity of the intestinal microflora, increasing the level of helpful bacteria, and reducing the level of harmful ones.

Furthermore, serum metabolomics was used to characterize serum metabolic profiles and screen differential metabolites between T2DM mice and AAL-treated groups. According to the screening criteria, 48 metabolites differed significantly between the AAL and model groups. Metabolomic analysis showed that the increase in L-Phenylalanine in T2DM mice was in line with the previous report that the branched-chain amino acid-related factor was significantly positively associated with insulin resistance [92]. L-phenylalanine is an aromatic amino acid whose elevated level increases the risk of T2DM and has a positive association with insulin resistance, hyperglycemia, and hyperlipidemia [93,94]. According to our results, AAL markedly reduced the level of the intermediate metabolite L-phenylalanine, relating to pathways involving phenylalanine, tyrosine and tryptophan biosynthesis, and phenylalanine metabolism, indicating that AAL improved T2DM through modulating amino acid metabolism disorders. Meanwhile, there is a significant positive correlation between L-phenylalanine and Firmicutes, and a negative correlation with Bacteroidetes and Bifidobacterium, indicating that the altered gut microbiota in T2DM, especially the increase in Firmicutes and depletion of Bacteroidetes, might be linked to the higher serum level of L-phenylalanine. Lipid metabolism is tightly associated with the mechanisms of the T2DM metabolic pathway [95]. Arachidonic acid metabolism and α-linoleic acid metabolism are two of the lipid metabolism pathways that have been identified in this study. Arachidonic acid (AA) is a polyunsaturated fatty acid, and numerous investigations have found that AA levels in the blood of T2DM mice were notably higher to promote the development of T2DM [96,97]. In the current research, the levels of serum AA and its pro-inflammatory metabolites, leukotriene B4, thromboxane B2, and 12-keto-leukotriene B4, were increased in T2DM mice, whereas the serum level of its anti-inflammatory metabolite, lipoxin A4, was decreased, which was in accordance with the previous research [95]. The serum levels of metabolites related to arachidonic acid metabolism were reversed following AAL intervention, thereby improving its metabolic disorders. In addition, AAL intervention increased the alpha-linolenic acid level, which is associated with lipid metabolism. Alpha-linolenic acid is an omega-3 fatty acid, and studies have discovered that omega-3 fatty acids reduce the risk of type 2 diabetes in the Chinese population [97]. Lysophosphatidylcholine is a class of bioactive lipids, and decreased levels of it are found in diabetes [98]. This was also confirmed in our research, which discovered that serum levels of several lysoPCs (LysoPC (P-18:1), LysoPC (22:6), LysoPC (20:4), and LysoPC (18:2)) were lower in the T2DM group and increased after AAL intervention.

## 5. Conclusions

In conclusion, this study outlined the hypoglycemic effect of AAL using UPLC-MS^E^-based metabolomics combined with gut microbiota 16S rRNA sequencing. AAL could alleviate T2DM-related symptoms and had a beneficial regulatory effect on intestinal flora structure and metabolite-related metabolic pathways. These results indicate that AAL might have certain potential in preventing and protecting against T2DM, providing a theoretical foundation for the resource utilization of AAL and a new way to achieve high value-added utilization. However, the gut microbial community composition at the species level and the metabolic pathway of the species composition needs to be further analyzed. Additionally, we speculated that the hypoglycemic effect of AAL might be responsible for its rich polyphenols. Therefore, further investigation is required to evaluate the relationship between transitional components and hypoglycemic effects, and elucidate the material basis of AAL against diabetes.

## Data Availability

All data generated or analyzed during this study are included in this published article (and its Supplementary Materials file).

## Supplementary Materials

The following are available online at https://www.mdpi.com/article/10.3390/nu15194115/s1, Figure S1. The separation workflow of AAL. Figure S2. Structure of compounds **1**–**25** from AAL. Figure S3. The HPLC chromatogram of AAL sample. Figure S4. The ratio of Firmicutes to Bacteroidetes (F/B). Figure S5. The PCA score plots in positive and negative modes. Figure S6. The permutation test plots of the OPLS-DA model in positive and negative modes. Table S1 The quantification of 25 compounds from the extract of AAL. Table S2. Identification results of potential metabolic markers in serum. Table S3. Metabolic pathways of differential metabolites.

## Abbreviations

T2DM: type 2 diabetes mellitus; AAL: *Actinidia arguta* leaves; STZ: streptozotocin; HFD: high-fat diet; BW: body weight; FBG: fasting blood glucose; INS: insulin; IR: insulin resistance; HOMA-IR: homeostasis model assessment: insulin resistance; OGTT: oral glucose tolerance test; ROS: reactive oxygen species; GSH-Px: glutathione peroxidase; SOD: superoxide dismutase; MDA: malondialdehyde; TG: triglycerides; TC: total cholesterol; ALT: alanine aminotransferase; AST: aspartate aminotransferase; Cre: creatinine; BUN: blood urea nitrogen; BUA: blood uric acid; PCoA: principal coordinates analysis; NMDS: non-metric multidimensional scaling; AA: arachidonic acid.
