# Supplementary material for: Gut Microbiota Combined with Serum Metabolomics to Investigate the Hypoglycemic Effect of Actinidia arguta Leaves
Source: Nutrients. 2023 Sep 23;15(19):4115. doi: 10.3390/nu15194115 (PMC10574697; doi:10.3390/nu15194115)
Supplement: Supplementary file 1 [file nutrients-15-04115-s001.zip › nutrients-2613039-supplementary.pdf]

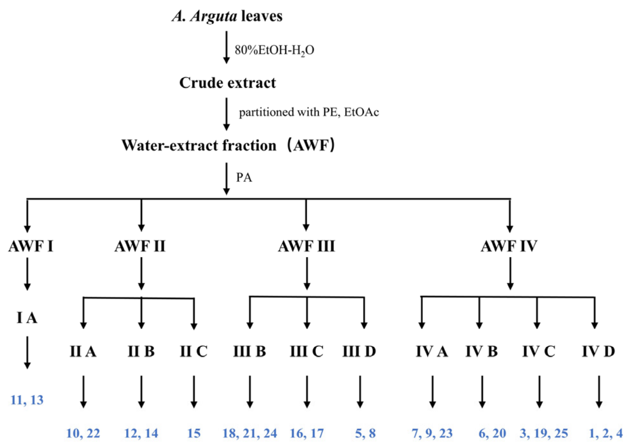

Figure S1 The separation workflow of AAL.

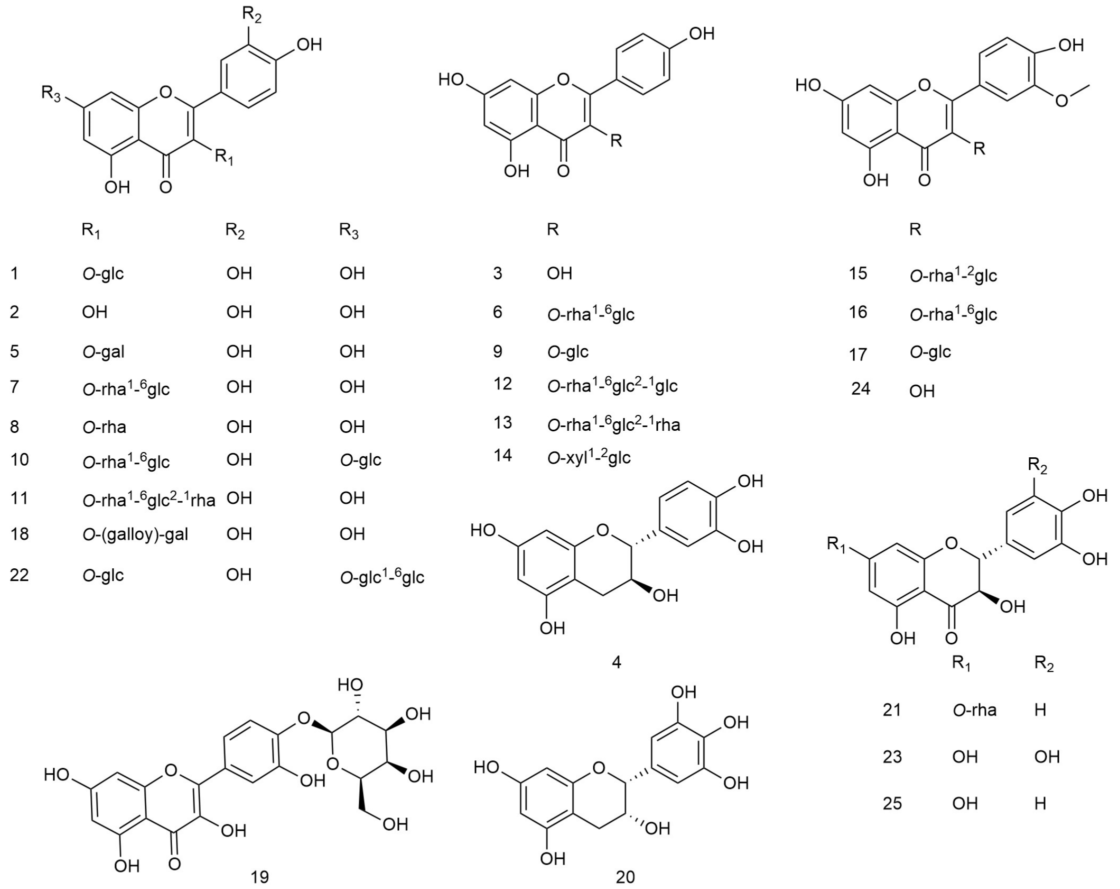

Figure S2 Structure of compounds **1 – 25** from AAL.

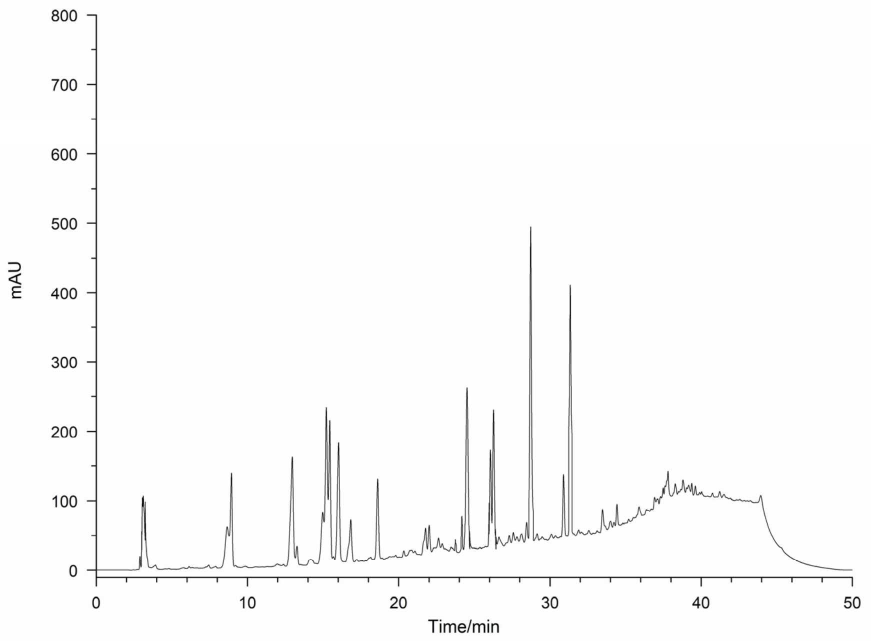

Figure S3 The HPLC chromatogram of AAL sample.

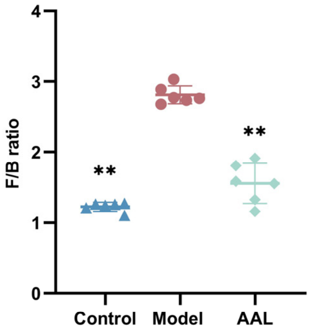

Figure S4. The ratio of Firmicutes to Bacteroidetes (F/B).

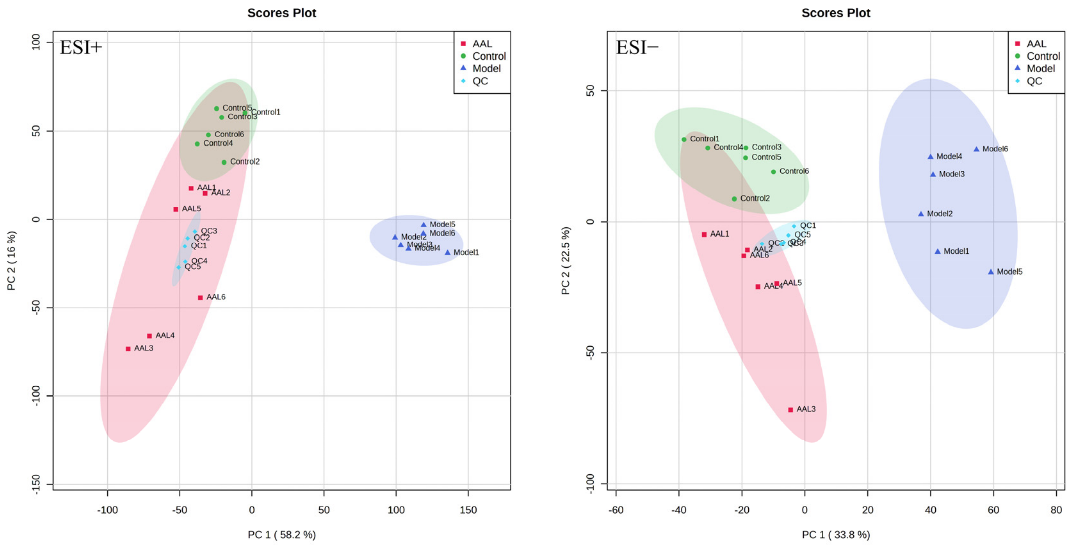

Figure S5. The PCA score plots in positive and negative modes.

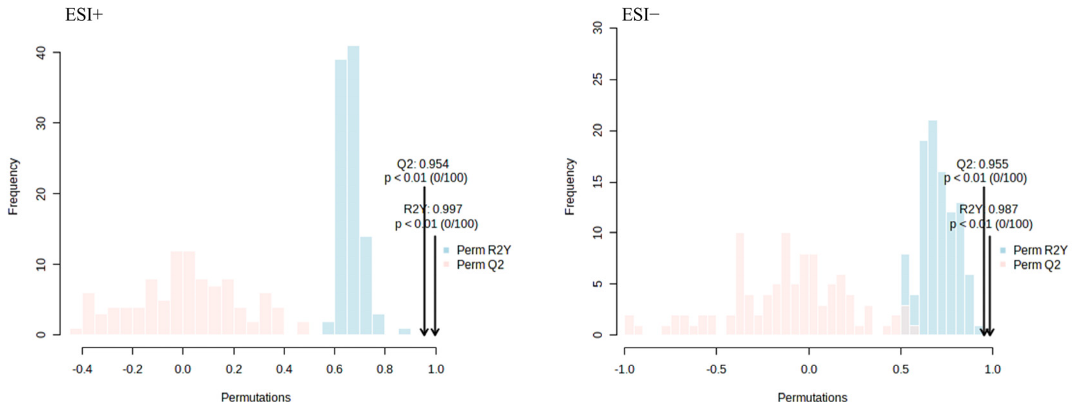

Figure S6. The permutation test plots of the OPLS-DA model in positive and negative modes.

Table S1 The quantification of 25 compounds from the extract of AAL.

Compounds	Calibration curves	R^2	LOD (μg/mL)	LOQ (μg/mL)	Content of compounds （mg/g DW, n=3）
1	y = 40661x + 365.24	0.9991	1.25	3.80	1.26 ± 0.01
2	y = 41847x + 335.15	0.9993	0.24	0.73	8.97 ± 0.09
3	y = 13528x + 65.33	0.9998	0.82	2.50	6.89 ± 0.04
4	y = 10723x + 112.89	0.9996	0.75	2.28	3.30 ± 0.03
5	y = 38042x + 305.8	0.9992	0.52	1.60	0.61 ± 0.01
6	y = 26187x + 101.08	0.9999	0.53	1.62	0.64 ± 0.01
7	y = 46001x − 286.35	0.9996	0.55	1.67	3.99 ± 0.01
8	y = 33782x + 152.87	0.9998	0.54	1.64	0.59 ± 0.01
9	y = 18641x + 115.61	0.9997	0.28	0.85	1.49 ± 0.01
10	y = 10430x − 14.457	0.9999	2.02	6.24	0.59 ± 0.01
11	y = 10564x − 14.432	0.9999	0.02	0.60	0.98 ± 0.01
12	y = 8531.3x + 40.423	0.9995	0.28	0.85	0.14 ± 0.002
13	y = 7713.1x + 90.29	0.9995	1.05	3.18	0.21 ± 0.002
14	y = 7797.7x + 110.98	0.9992	2.34	7.12	0.22 ± 0.005
15	y = 8633.9x + 134.5	0.9993	0.83	2.53	0.23 ± 0.01
16	y = 5758.9x + 85.676	0.9991	1.35	4.15	0.19 ± 0.005
17	y = 1912.1x + 108.18	0.9994	0.25	0.75	1.43 ± 0.01
18	y = 26990x − 102.89	0.9998	1.05	3.25	1.36 ± 0.02
19	y = 39977x − 369.66	0.9994	1.55	4.70	0.63 ± 0.005
20	y = 17158x + 202.80	0.9991	0.81	2.48	1.38 ± 0.01
21	y = 3864.7x + 54.761	0.9991	0.55	1.68	0.80 ± 0.01
22	y = 6896.5x + 66.603	0.9996	0.52	1.58	0.24 ± 0.01
23	y = 18789x + 186.93	0.9993	0.82	2.50	1.60 ± 0.02
24	y = 61202x + 475.2	0.9991	0.54	1.65	2.38 ± 0.01
25	y = 8156x + 34.512	0.9998	1.08	3.30	0.89 ± 0.01

Table S2 Identification results of potential metabolic markers in serum.

No.	t_R /min	Measured mass	Formula	Adducts	Mass Error (ppm)	Metabolites	Compound ID	VIP	Trends（AAL/Model）
1	18.07	401.3402*m/z*	$C_{27}H_{44}O_2$	M+H	−3.02	7α-Hydroxy-cholestene-3-one	HMDB0001993	1.71	↓(**)
2	15.72	506.3604*m/z*	$C_{26}H_{52}NO_6P$	M+H	−0.13	LysoPC(P-18:1(9Z))	HMDB0010408	1.34	↑(**)
3	9.44	309.2045*m/z*	$C_{16}H_{30}O_4$	M+Na	3.19	Hexadecanedioic acid	HMDB0000672	1.67	↓(**)
4	7.04	405.2645*m/z*	$C_{24}H_{38}O_5$	M−H	−0.45	7-Ketodeoxycholic acid	HMDB0000391	1.41	↓(**)
5	8.22	291.1964*m/z*	$C_{18}H_{28}O_3$	M−H	−0.43	(2'E,4'Z,7'Z,8E)-Colnelenic acid	HMDB0030996	1.40	↓(*)
6	14.33	400.3410*m/z*	$C_{23}H_{45}NO_4$	M+H	−2.93	L-Palmitoylcarnitinel	HMDB0000222	1.72	↓(**)
7	11.35	567.3334n	$C_{30}H_{50}NO_7P$	M+Na, 2M+H, M+H	1.61	LysoPC(22:6(4Z,7Z,10Z,13Z,16Z,19Z))	HMDB0010404	1.54	↑(**)
8	11.17	544.3398*m/z*	$C_{28}H_{50}NO_7P$	M+H	0.12	LysoPC(20:4(5Z,8Z,11Z,14Z))	HMDB0010395	1.51	↑(*)
9	8.26	307.1885*m/z*	$C_7H_{11}N_3O$	2M+H	2.49	N-Acetylhistaminen	HMDB0013253	1.50	↑(*)
10	5.75	512.2687*m/z*	$C_{26}H_{43}NO_7S$	M−H	−0.01	Sulfolithocholylglycine	HMDB0002639	1.39	↓(*)
11	12.54	295.2250*m/z*	$C_{18}H_{32}O_3$	M−H	−0.84	13S-hydroxyoctadecadienoic acid	HMDB0004667	1.38	↓(**)
12	10.7	392.2926n	$C_{24}H_{40}O_4$	M−H, M+Cl	−0.03	Chenodeoxycholic acid	HMDB0000518	1.36	↓(**)
13	10.7	392.2926n	$C_{24}H_{40}O_4$	M−H, M+Cl	−0.03	Deoxycholic acid	HMDB0000626	1.36	↓(**)
14	10.7	392.2926n	$C_{24}H_{40}O_4$	M−H, M+Cl	−0.03	Ursodeoxycholic acid	HMDB0000946	1.36	↓(**)
15	16.24	340.2832*m/z*	$C_{18}H_{39}NO_3$	M+Na	3	Phytosphingosine	HMDB0004610	1.43	↑(*)
16	4.79	188.0690*m/z*	$C_9H_{11}NO_2$	M+Na	4.94	L-Phenylalanine	HMDB0000159	1.51	↓(**)
17	2.15	192.0270n	$C_6H_8O_7$	M−H_2O−H, M−H	−0.17	Citric acid	HMDB0000094	1.36	↑(**)
18	2.15	192.0270n	$C_6H_8O_7$	M−H_2O−H, M−H	−0.18	Isocitric acid	HMDB0000193	1.35	↑(*)
19	7.83	355.2622*m/z*	$C_{22}H_{36}O_2$	M+Na	4.49	Adrenic acid	HMDB0002226	1.69	↓(**)
20	7.9	408.2874n	$C_{24}H_{40}O_5$	M−H, M+Cl	−0.42	Cholic acid	HMDB0000619	1.34	↓(**)
21	9.35	295.2276*m/z*	$C_{16}H_{32}O_3$	M+Na	2.33	16-Hydroxy hexadecanoic acid	HMDB0006294	1.72	↓(*)
22	5.69	515.2927n	$C_{26}H_{45}NO_7S$	M+Na, 2M+H, M+H	2.02	Taurocholic acid	HMDB0000036	1.71	↓(**)
23	13.84	277.2145*m/z*	$C_{16}H_{30}O_2$	M+Na	2.61	Palmitoleic acid	HMDB0003229	1.60	↓(*)

24	5.25	356.1642*m/z*	$C_{14}H_{23}N_6O_3S$	M+H	4.79	S-Adenosylmethioninamines	HMDB0000988	1.54	↑(*)
25	14.66	279.2305*m/z*	$C_{16}H_{32}O_2$	M+Na	3.97	Palmitic acid	HMDB0000220	1.52	↓(**)
26	7.42	311.2201*m/z*	$C_{16}H_{32}O_4$	M+Na	2.73	(S)-10,16-Dihydroxyhexadecanoic acid	HMDB0037798	1.49	↓(**)
27	16.66	335.2931*m/z*	$C_{20}H_{40}O_2$	M+Na	3.24	Phytanic acid	HMDB0000801	1.46	↓(*)
28	1.72	215.0329*m/z*	$C_6H_{12}O_6$	M+Cl	0.37	D-Mannose	HMDB0000169	1.33	↓(**)
29	1.72	215.0329*m/z*	$C_6H_{12}O_6$	M+Cl	0.37	myo-Inositol	HMDB0000211	1.33	↓(**)
30	1.65	258.1089*m/z*	$C_8H_{20}NO_6P$	M+H	−4.81	Glycerophosphocholine	HMDB0000086	1.53	↑(*)
31	7.07	369.2280*m/z*	$C_{20}H_{34}O_6$	M−H	−0.77	Thromboxane B2	HMDB0003252	1.33	↓(**)
32	14.66	279.2305*m/z*	$C_{18}H_{30}O_2$	M+H	−4.99	Alpha-Linolenic acid	HMDB0001388	1.52	↑(*)
33	14.66	279.2305*m/z*	$C_{18}H_{30}O_2$	M+H	−4.99	Gamma-Linolenic acid	HMDB0003073	1.51	↓(**)
34	10.05	336.2296n	$C_{20}H_{32}O_4$	M−H2O−H, M−H	−1.30	Leukotriene B4	HMDB0001085	1.31	↓(**)
35	10.4	333.2108*m/z*	$C_{19}H_{28}O_2$	M+FA−H	−0.35	Dehydroepiandrosterone	HMDB0000077	1.34	↑(*)
36	6.06	289.1816*m/z*	$C_{18}H_{24}O_3$	M+H	−4.84	2-Hydroxyestradio	HMDB0000338	1.51	↑(*)
37	10.4	333.2070*m/z*	$C_{20}H_{30}O_4$	M−H	−0.31	12-Keto-leukotriene B4	HMDB0004234	1.30	↓(*)
38	10.4	333.2001*m/z*	$C_{20}H_{32}O_5$	$M-H_2O-H$	−0.28	Lipoxin A4	HMDB0004385	1.34	↑(*)
39	12.98	327.2305*m/z*	$C_{20}H_{32}O_2$	M+Na	3.58	Arachidonic acid	HMDB0001043	1.70	↓(**)
40	9.52	301.2149*m/z*	$C_{20}H_{28}O_2$	M+H	−4.29	all-trans-Retinoic acid	HMDB0001852	1.69	↓(**)
41	9.52	301.2148*m/z*	$C_{20}H_{28}O_2$	M+H	−4.29	9-cis-Retinoic acid	HMDB0002369	1.68	↓(**)
42	10.36	407.2395*m/z*	$C_9H_{17}NO_4$	2M+H	1.65	L-Acetylcarnitine	HMDB0000201	1.66	↓(*)
43	8.63	520.3402*m/z*	$C_{26}H_{50}NO_7P$	M+H	0.77	LysoPC(18:2(9Z,12Z))	HMDB0010386	1.48	↑(*)
44	5.22	522.2837*m/z*	$C_{26}H_{45}NO_6S$	M+Na	−4.55	Taurodeoxycholic acid	HMDB0000896	1.60	↓(**)
45	5.22	522.2839*m/z*	$C_{26}H_{45}NO_6S$	M+Na	−4.55	Taurochenodesoxycholic acid	HMDB0000951	1.49	↓(**)
46	9.59	367.2467*m/z*	$C_{21}H_{34}O_5$	M+H	−3.20	Tetrahydrocortisol	HMDB0000949	1.72	↑(*)
47	1.86	365.1042*m/z*	$C_{12}H_{22}O_{11}$	M+Na	−3.55	Trehalose	HMDB0000975	1.54	↓(**)
48	1.86	365.1044*m/z*	$C_{12}H_{22}O_{11}$	M+Na	−3.55	Glucose-1,3-mannose oligosaccharide	HMDB0060068	1.34	↑(*)

Table S3 Metabolic pathways of differential metabolites.

Metabolism	Pathway	Total	Hits	Raw p	-log10(P)	Holm adjust	FDR	Impact
Amino acid metabolism	Phenylalanine, tyrosine and tryptophan biosynthesis	4	1	0.06	1.21	1	0.91	0.50
	Phenylalanine metabolism	10	1	0.14	0.83	1	1.00	0.35
Lipid metabolism	Arachidonic acid metabolism	36	2	0.10	0.97	1	0.91	0.33
	alpha-Linolenic acid metabolism	13	1	0.18	0.73	1	1.00	0.33
Metabolism of cofactors and vitamins	Retinol metabolism	17	2	0.02	1.56	1	0.76	0.21
Carbohydrate metabolism	Citrate cycle (TCA cycle)	20	2	0.03	1.43	1	0.77	0.13

NMR data:

Compound 1: Isoquercitrin

^{13}C NMR (151 MHz, CD_3OD-d_4) δ 158.5(C-2), 135.6 (C-3), 179.5 (C-4), 163.1 (C-5), 99.9 (C-6), 166.0 (C-7), 94.7 (C-8), 158.9 (C-9), 105.7 (C-10), 123.1 (C-1'), 117.5 (C-2'), 145.9 (C-3'), 149.9 (C-4'), 116.0 (C-5'), 123.2 (C-6'),104.3 (C-1''), 73.1 (C-2''), 75.7 (C-3''), 71.2 (C-4''), 78.1 (C-5''), 62.5 (C-6''). ^{1}H NMR (600 MHz, CD_3OD-d_4) δ 6.36 (1H, d, J = 2.1 Hz, H-8), 6.17 (1H, d, J = 2.3 Hz, H-6), 5.23 (1H, d, J = 7.7 Hz, H-1''), 3.69 (1H, dd, J = 11.9, 2.3 Hz, H-6''a), 3.55 (1H, dd, J = 11.9, 5.4 Hz, H-6''b), 3.47 – 3.37 (2H, m, H-2'', H-3''), 3.34 – 3.30 (1H, m, H-4''), 3.20 (1H, ddd, J = 9.9, 5.4, 2.4 Hz, H-5'').

Compound 2: Quercetin

^{13}C-NMR (150 MHz, C_5D_5N-d_5) δ 147.5 (C-2), 137.7 (C-3), 177.1 (C-4), 157.2 (C-5), 99.0 (C-6), 165.3 (C-7), 94.1 (C-8), 162.2 (C-9), 104.2 (C-10), 123.7 (C-1'), 116.2 (C-2'), 146.9 (C-3'), 149.3 (C-4'), 116.4 (C-5'), 120.8 (C-6'). ^{1}H-NMR (600 MHz, C_5D_5N-d_5) δ 6.56 (1H, d, J = 8.4 Hz, H-8), 6.36 (1H, d, J = 1.4 Hz, H-6), 7.78 (1H, d, J = 2.2 Hz, H-2'), 7.26 (1H, dd, J = 8.4, 2.2 Hz, H-6'), 6.56 (1H, d, J = 8.4 Hz, H-5').

Compound 3: Kaempferol

^{13}C-NMR (150 MHz, CD_3OD-d_4) δ 146.7 (C-2), 135.9 (C-3), 176.1 (C-4), 156.9 (C-5), 97.9 (C-6), 164.2 (C-7), 93.7 (C-8), 161.2 (C-9), 103.2 (C-10), 122.4 (C-1'), 129.3 (C-2', 6'), 115.0 (C-3', 5'). ^{1}H NMR (600 MHz, CD_3OD-d_4) δ 8.06 (2H, d, J = 8.9 Hz, H-2', 6'), 6.87 (2H, d, J = 8.9 Hz, H-3', 5'), 6.37 (1H, d, J = 1.6 Hz, H-8), 6.15 (1H, s, H-6).

Compound 4: Catechin

^{13}C-NMR (151 MHz, CD_3OD-d_4) δ 81.5 (C-2), 67.5 (C-3), 27.2 (C-4), 156.5 (C-5), 95.0 (C-6), 156.2 (C-7), 94.2 (C-8), 155.6 (C-9), 99.5 (C-10), 130.9 (C-1'), 114.8 (C-2'), 144.9 (C-3', 4'), 113.9 (C-5'), 118.7 (C-6'). ^{1}H-NMR (600 MHz, CD_3OD-d_4) δ 4.54 (1H, d, J = 7.5 Hz, H-2), 3.95 (1H, m, H-3), 2.82 (1H, dd, J = 16.1, 5.4 Hz, H-4a), 2.48 (1H, dd, J = 16.1, 8.2 Hz, H-4b), 6.81 (1H, d, J = 2.0 Hz, H-2'), 6.74 (1H, d, J = 8.1 Hz, H-5'), 6.69 (1H, dd, J = 8.6, 2.1 Hz, H-6'), 5.90 (1H, d, J = 2.3 Hz, H-8), 5.83 (1H, d, J = 2.3 Hz, H-6).

Compound 5: Hyperoside

^{13}C-NMR (150 MHz, C_5D_5N-d_5) δ 157.6 (C-2), 135.7 (C-3), 178.6 (C-4), 162.5 (C-5), 99.5 (C-6), 165.7 (C-7), 94.3 (C-8), 157.3 (C-9), 104.9 (C-10), 122.1 (C-1'), 116.0 (C-2'), 146.5 (C-3'), 150.5

(C-4'), 117.2 (C-5'), 122.6 (C-6'), 105.3 (C-1''), 73.2 (C-2''), 75.2 (C-3''), 69.5 (C-4''), 77.5 (C-5''), 61.7 (C-6''). ^{1}H-NMR (600 MHz, C_5D_5N-d_5) δ 8.38 (1H, d, J = 2.2 Hz, H-2'), 8.05 (1H, dd, J = 8.3, 2.2 Hz, H-6') 7.18 (1H, d, J = 8.4 Hz, H-5'), 6.61 (1H, d, J = 2.1 Hz, H-8), 6.56 (1H, d, J = 2.1 Hz, H-6), 6.01 (1H, d, J = 7.8 Hz, H-1''), 4.75 – 4.11 (6H, m, other galactosyl proton signal).

Compound 6: Nicotiflorin

^{13}C NMR (151MHz, CD_3OD-d_4) δ 157.2(C-2), 134.1 (C-3), 178.0 (C-4), 161.6 (C-5), 98.6 (C-6), 164.6 (C-7), 93.5 (C-8), 158.0 (C-9), 103.2 (C-10), 121.4 (C-1'), 131.0 (C-2', 6'), 114.7 (C-3',5'), 160.1 (C-4'), 104.3 (C-1''), 74.4(C-2''), 76.8(C-3''), 70.9(C-4''), 75.8(C-5''), 67.2(C-6''), 101.1(C-1'''), 70.7(C-2'''), 70.1(C-3'''),72.5 (C-4'''), 68.3(C-5'''), 16.5(C-6'''). ^{1}H-NMR (600 MHz, CD_3OD-d_4) δ 8.04 (2H, d, J = 8.9 Hz, H-2', 6'), 6.87 (2H, d, J = 9.0 Hz, H-3'. 5'), 6.38 (1H, d, J = 2.1 Hz, H-8), 6.19 (1H, d, J = 2.1 Hz, H-6), 5.11 (1H, d, J = 7.5 Hz, H-1''), 4.49 (1H, d, J = 1.8 Hz, H-1''').

Compound 7: Rutin

^{13}C NMR (151 MHz, C_5D_5N-d_5) δ 158.0 (C-2), 135.7 (C-3), 178.4 (C-4), 162.5 (C-5), 99.6 (C-6), 165.7 (C-7), 94.4 (C-8), 157.5 (C-9), 105.0 (C-10), 122.9 (C-1'), 117.7 (C-2'), 146.6 (C-3'), 149.3 (C-4'), 116.1 (C-5'), 123.7 (C-6'), 104.7 (C-1''), 75.9 (C-2''), 78.5 (C-3''), 71.1 (C-4''), 77.3 (C-5''), 68.3 (C-6''), 102.4 (C-1'''), 71.9 (C-2'''), 72.4 (C-3'''), 73.8 (C-4'''), 69.4 (C-5'''), 18.3 (C-6'''). ^{1}H NMR (600 MHz, C_5D_5N-d_5) δ 8.29 (1H, d, J = 2.1 Hz,H-2'), 8.05 (1H, dd, J = 8.4, 2.2 Hz, H-6'), 7.29 (1H, d, J = 8.4 Hz,H-5'), 6.61 (1H, d, J = 2.1 Hz, H-8), 6.57 (1H, d, J = 2.1 Hz, H-6),5.94 (1H , d, J = 7.5 Hz, H-1''), 5.29 (1H, s, H-1'''), 1.45 (3H , d, J = 6.0 Hz, H-6''').

Compound 8: Quercitrin

^{13}C NMR (151 MHz, CD_3OD-d_4) δ 157.9 (C-2), 134.9 (C-3), 178.3 (C-4), 157.2 (C-5), 96.4 (C-6), 164.5 (C-7), 93.3 (C-8), 161.9 (C-9), 104.5 (C-10), 121.5 (C-1'), 115.5 (C-2'), 145.0 (C-3'), 148.4 (C-4'), 115.0 (C-5'), 121.6 (C-6'), 102.2 (C-1''), 70.6 (C-2''), 70.9 (C-3''), 70.5 (C-4''), 70.7 (C-5''), 16.3 (C-6''). ^{1}H NMR (600 MHz, CD_3OD-d_4) δ 7.31 (1H, d, J = 2.1 Hz, H-2'), 7.28 (1H, dd, J = 8.3, 2.1 Hz, H-6'), 6.88 (1H, d, J = 8.4 Hz, H-5'), 6.35 (1H, d, J = 2.1 Hz, H-8), 6.18 (1H, d, J = 2.3 Hz, H-6), 5.32 (1H, d, J = 1.6 Hz, H-1″), 0.91 (3H, d, J = 6.2 Hz, H-6''), 4.19 (1H, dd, J = 3.4, 1.7 Hz, H-2''), 3.72 (1H , dd, J = 9.5, 3.4 Hz, H- 3''), 3.40 (1H, m, H- 5''), 3.29 (1H, m, H- 4'').

Compound 9: Astragalin

^{13}C-NMR (151 MHz, C_5D_5N-d_5) δ 157.9 (C-2), 135.4 (C-3), 179.1 (C-4), 157.8 (C-5), 100.2 (C-6), 167.2 (C-7), 95.0(C-8), 163.2 (C-9), 105.6 (C-10), 122.3 (C-1'), 132.2 (C-2', 6'), 116.4 (C-3', 5'),

162.0 (C-4'), 104.3 (C-1''), 76.3 (C-2''), 78.8 (C-3''), 71.8 (C-4''), 79.4 (C-5''), 62.9 (C-6''). ^{1}H-NMR (600 MHz, C_5D_5N-d_5) δ 8.37 (2H, d, J = 8.8 Hz, H-2', 6'), 7.13 (2H, d, J = 8.7 Hz, H-3', 5'), 6.64 (1H, d, J = 0.7 Hz, H-8), 6.28 (1H, d, J = 7.5 Hz, H-6).

Compound 10: Quercetin-3-*O*-rutinoside-7-*O*-glucoside

^{13}C-NMR (101 MHz, C_5D_5N-d_5) δ 158.9 (C-2), 135.1 (C-3), 178.6 (C-4), 162.2 (C-5), 100.9 (C-6), 164.0 (C-7), 95.2 (C-8), 157.1 (C-9), 106.9 (C-10), 122.4 (C-1'), 118.2 (C-2'), 146.9 (C-3'), 150.5 (C-4'), 116.4 (C-5'), 123.1 (C-6'), 104.6 (C-1''), 76.1 (C-2''), 79.3 (C-3''), 71.4 (C-4''), 77.7 (C-5''), 68.5 (C-6''), 102.4 (C-1'''), 72.2 (C-2'''), 72.6 (C-3'''), 74.0 (C-4'''), 69.7 (C-5'''), 18.6 (C-6'''), 101.7 (C-1''''), 74.9 (C-2''''), 78.5 (C-3''''), 71.2 (C-4''''), 78.7 (C-5''''), 62.5 (C-6''''). ^{1}H-NMR (400 MHz, C_5D_5N-d_5) δ 8.35 (1H, d, J = 2.6 Hz, H-2'), 8.07 (1H, dd, J = 8.3, 2.7 Hz, H-6'), 7.32 (1H, d, J = 8.5 Hz, H-5'), 6.89 (1H ,d, J = 2.7 Hz, H-8), 6.74 (1H, d, J = 2.5 Hz, H-6), 6.02 (1H, d, J = 7.4 Hz, H-1''), 5.79 (1H, d, J = 7.7 Hz, H-1'''), 5.32 (1H, s, H-1''''), 1.50 (3H, d, J = 4.6 Hz, H-6''').

Compound 11: Manghaslin

^{13}C-NMR (101 MHz, CD_3OD-d_4) δ 158.9 (C-2), 133.7 (C-3), 179.3 (C-4), 163.2 (C-5), 99.7 (C-6), 165.6 (C-7), 94.5 (C-8), 158.6 (C-9), 105.9 (C-10), 123.4 (C-1'), 117.4 (C-2'), 145.9 (C-3'), 149.6 (C-4'), 116.0 (C-5'), 123.3 (C-6'), 100.5 (C-1''), 80.0 (C-2''), 78.9 (C-3''), 71.8 (C-4''), 77.2 (C-5''), 68.3 (C-6''), 102.3 (C-1'''), 72.1 (C-2'''), 72.2 (C-3'''), 74.0 (C-4'''), 69.8 (C-5'''), 17.8 (C-6'''), 102.7 (C-1''''), 72.3 (C-2''''), 72.3 (C-3''''), 73.8 (C-4''''), 69.9 (C-5''''), 17.5 (C-6''''). ^{1}H-NMR (400 MHz, CD_3OD-d_4) δ7.62 (1H, d, J = 8.1 Hz, H-2'), 7.13 (1H, d, J = 5.9 Hz, H-6'), 6.94 (1H, d, J = 5.9 Hz, H-5'), 6.34 (1H, d, J = 6.8 Hz, H-8), 5.97 (1H, s, H-6), 5.39 (1H, s, H-1''), 5.26 (1H, s, H-1'''), 4.56 (1H, s, H-1'''').

Compound 12: Kaempferol-3-*O*-(2''-*O*-β-D-glucopyl)-β-D-rutinoside

^{13}C-NMR (101 MHz, CD_3OD-d_4) δ 156.7 (C-2), 132.8 (C-3), 177.6 (C-4), 161.8 (C-5), 99.3 (C-6), 164.5 (C-7), 93.8 (C-8), 156.4 (C-9), 102.2 (C-10), 121.0 (C-1'), 130.5 (C-2', 6'), 114.4 (C-3', 5'), 159.5 (C-4'), 98.6 (C-1''), 82.0 (C-2''), 75.9 (C-3''), 69.3 (C-4''), 75.6 (C-5''), 65.9 (C-6''), 100.3 (C-1'''), 70.1 (C-2'''), 69.4 (C-3'''), 71.9 (C-4'''), 68.0 (C-5'''), 16.5 (C-6'''), 102.5 (C-1''''), 74.8 (C-2''''), 76.3 (C-3''''), 70.3 (C-4''''), 77.6 (C-5''''), 60.6 (C-6''''). ^{1}H-NMR (400 MHz, CD_3OD-d_4) δ 8.64 (2H, d, J = 8.1 Hz, H-2', 6'), 7.53 (2H, d, J = 8.0 Hz, H-3', 5'), 7.02 (1H, s, H-6), 6.85 (1H, s, H-8), 5.90 (1H, d, J = 7.3 Hz, H-1''), 5.38 (1H, d, J = 6.5 Hz, H-1''''), 5.09 (1H, s, H-1'''), 1.72 (3H, m, H-6''').

Compound 13: Clitorin

^{13}C-NMR (101 MHz, CD_3OD-d_4) δ 155.9 (C-2), 132.3 (C-3), 177.6 (C-4), 161.3 (C-5), 98.5 (C-6), 163.8 (C-7), 93.6 (C-8), 156.8 (C-9), 104.8 (C-10), 121.0 (C-1'), 130.8 (C-2'), 115.6 (C-3'), 159.1 (C-4'), 115.0 (C-5'), 130.2 (C-6'), 98.0 (C-1''), 75.9 (C-2''), 77.6 (C-3''), 70.8 (C-4''), 77.3 (C-5''), 68.1 (C-6''), 100.6 (C-1'''), 70.3 (C-2'''), 70.5 (C-3'''), 71.7 (C-4'''), 68.8 (C-5'''), 17.5 (C-6'''), 100.5 (C-1''''), 69.9 (C-2''''), 70.9 (C-3''''), 71.0 (C-4''''), 68.5 (C-5''''), 17.0 (C-6''''). ^{1}H NMR (400 MHz, CD_3OD-d_4) δ 7.69 (2H, d, J = 8.5 Hz, H-2', 6'), 7.41 (2H, d, J = 9.1 Hz, H-3', 5'), 6.15 (1H, s, H-8), 5.84 (1H, s, H-6), 4.78 (1H, s, H-1''), 4.64 (1H, s, H-1'''), 4.50 (1H, s, H-1'''').

Compound 14: Leucoside

^{13}C-NMR (101 MHz, CD_3OD-d_4) δ 161.5 (C-2), 135.7 (C-3), 179.6 (C-4), 163.8 (C-5), 100.6 (C-6), 165.6 (C-7), 94.6 (C-8), 158.7 (C-9), 105.5 (C-10), 123.4 (C-1'), 132.3 (C-2', 6'), 116.2 (C-3', 5'), 159.0 (C-4'), 100.7 (C-1''), 83.4 (C-2''), 75.0 (C-3''), 72.0 (C-4''), 79.1 (C-5''), 62.2 (C-6''), 105.3 (C-1'''), 78.2 (C-2'''), 78.4 (C-3'''), 71.1 (C-4'''), 66.8 (C-5'''). ^{1}H NMR (400 MHz, CD_3OD-d_4) δ 8.83 (2H, d, J = 7.5 Hz, H-2', 6'), 7.65 (2H, d, J = 8.0 Hz, H-3', 5'), 7.15 (1H, d, J = 2.1 Hz, H-8), 6.95 (1H, d, J = 2.9 Hz, H-6), 6.24 (1H, d, J = 7.0 Hz, H-1''), 5.38 (1H, s, H-1''').

Compound 15: Isorhamnetin-3-*O*-neohesperidin

^{13}C-NMR (101 MHz, CD_3OD-d_4) δ 158.2 (C-2), 134.3 (C-3), 179.3 (C-4), 163.2 (C-5), 99.7 (C-6), 165.7 (C-7), 94.6 (C-8), 158.4 (C-9), 106.0 (C-10), 123.5 (C-1'), 116.0 (C-2'), 148.3 (C-3'), 150.6 (C-4'), 114.5 (C-5'), 123.4 (C-6'), 56.9 (OCH_3), 100.6 (C-1''), 78.8 (C-2''), 78.4 (C-3''), 69.9 (C-4''), 80.3 (C-5''), 62.4 (C-6''), 102.8 (C-1'''), 72.3 (C-2'''), 72.4 (C-3'''), 73.9 (C-4'''), 71.8 (C-5'''), 17.4 (C-6'''). ^{1}H-NMR (400 MHz, CD_3OD-d_4) δ 7.94 (1H, d, J = 2.1 Hz, H-2'), 7.50 (1H, dd, J = 8.4, 2.1 Hz, H-6'), 6.88 (1H , d, J = 8.4 Hz, H-5'), 6.33 (1H, s, J = 2.2 Hz , H-8), 6.14 (1H , d, J = 2.3 Hz, H-6), 5.86 (1H , d, J = 6.3 Hz, H-1''), 5.14 (1H, s, H-1'''), 3.93 (3H, s, OCH_3), 0.82 (3H, d, J = 5.4 Hz, H-6''').

Compound 16: Narcissoside

^{13}C-NMR (101 MHz, CD_3OD-d_4) δ 158.4 (C-2), 135.4 (C-3), 179.3 (C-4), 163.0 (C-5), 99.9 (C-6), 166.0 (C-7), 94.9 (C-8), 158.8 (C-9), 105.6 (C-10), 123.9 (C-1'), 114.5 (C-2'), 148.3 (C-3'), 150.8 (C-4'), 116.1 (C-5'), 122.9 (C-6'), 56.7 (OCH_3), 104.4 (C-1''), 75.9 (C-2''), 78.1 (C-3''), 72.2 (C-4''), 77.3 (C-5''), 68.5 (C-6''), 102.5 (C-1'''), 72.0 (C-2'''), 71.6 (C-3'''), 73.8 (C-4'''), 69.8 (C-5'''), 17.9 (C-6'''). ^{1}H NMR (400 MHz, CD_3OD-d_4) δ 7.90 (1H, d, J = 2.1 Hz, H-2'), 7.57 (1H, dd, J = 8.5, 2.1 Hz,

H-6'), 6.87 (1H, d, *J* = 8.5 Hz, H-5'), 6.35 (1H ,d, J = 2.1 Hz, H-8), 6.16 (1H, d, J = 2.1 Hz, H-6), 5.20 (1H , d, *J* = 7.5 Hz, H-1''), 4.59 (1H, s, H-1'''), 3.90 (3H, s, OCH_3), 1.06 (3H, d, *J* = 6.2 Hz,H-6''').

Compound 17: Isorhamnetin-3-*O*-β-D-glucopyranosid

^{13}C-NMR (101 MHz, C_5D_5N-d_5) δ 157.5 (C-2), 134.5 (C-3), 178.6 (C-4), 162.9 (C-5), 99.7 (C-6), 165.8 (C-7), 94.5 (C-8), 157.1 (C-9), 105.2 (C-10), 122.0 (C-1'), 114.2 (C-2'), 149.2 (C-3'), 147.9 (C-4'), 116.1 (C-5'), 122.9 (C-6'), 56.0 (OCH_3), 103.2 (C-1''), 76.2 (C-2''), 79.0 (C-3''), 71.2 (C-4''), 78.4 (C-5''), 62.0 (C-6''). ^{1}H-NMR (400 MHz, C_5D_5N-d_5) δ 7.95 (1H, d, *J* = 2.0 Hz, H-2'), 7.63 (1H, dd, *J* = 8.1, 2.1 Hz, H-6'), 6.95 (1H, d, *J* = 8.1 Hz, H-5'), 6.73 (1H, d, *J* = 2.0 Hz, H-8), 5.96 (1H, d, *J* = 2.0 Hz, H-6), 3.98 (3H, s, OCH_3-3').

Compound 18: 2''-*O*-galloylhyperin

^{13}C-NMR (101 MHz, CD_3OD-d_4) δ 157.5 (C-2), 134.5 (C-3), 178.3 (C-4), 162.9 (C-5), 99.4 (C-6), 164.8 (C-7), 94.3 (C-8), 157.7 (C-9), 105.6 (C-10), 122.0 (C-1'), 115.8 (C-2'), 145.3 (C-3'), 149.0 (C-4'), 117.2 (C-5'), 122.9 (C-6'), 100.2 (C-1''), 74.2 (C-2''), 72.8 (C-3''), 70.1 (C-4''), 76.8 (C-5''), 61.7 (C-6''), 123.1 (C-1'''), 110.3 (C-2''', 6'''), 145.8 (C-3''', 5'''), 138.7 (C-4'''), 166.7 (C-7'''). ^{1}H NMR (400 MHz, CD_3OD-d_4) δ 7.80 (1H, d, *J* = 2.2 Hz, H-2'), 7.67 (1H, dd, *J* = 8.5, 2.2 Hz, H-6'), 7.21 (2H, s, H-2''',6'''), 6.89 (1H, d, *J* = 8.5 Hz, H-5'), 6.42 (1H, d, *J* = 2.1 Hz, H-8), 6.19 (1H, d, *J* = 2.1 Hz, H-6), 5.90 (1H, d, *J* = 8.0 Hz, H-1''), 5.46 (1H, dd, *J* = 9.7, 8.0 Hz, H-2''), 4.03 (1H, d, *J* = 3.4 Hz, H-4''), 3.96 (1H, dd, *J* = 9.8, 3.4 Hz, H-3''), 3.72 – 3.61 (3H, m, H-5'',6'').

Compound 19: Quercetin-4'-*O*-galactoside

^{13}C NMR (151 MHz, CD_3OD-d_4) δ 157.7 (C-2), 134.3 (C-3), 178.2 (C-4), 161.7 (C-5), 98.5 (C-6), 164.7 (C-7), 93.4 (C-8), 157.1 (C-9), 104.4 (C-10), 121.9 (C-1'), 114.7 (C-2'), 144.6 (C-3', 5'), 132.3 (C-4'), 116.2 (C-), 121.7 (C-6'), 102.9 (C-1''), 71.8 (C-2''), 74.4 (C-3''), 69.9 (C-4''), 76.8 (C-5''), 61.2 (C-6''). ^{1}H NMR (600 MHz, CD_3OD-d_4) δ 7.68 (d, *J* = 2.2 Hz, 1H, H-2'), 7.56 (1H, dd, *J* = 8.5, 2.2 Hz, H-6'), 6.84 (1H, d, *J* = 8.5 Hz, H-5'), 6.37 (1H, d, *J* = 2.1 Hz, H-8), 6.18 (1H, d, *J* = 2.0 Hz, H-6), 5.23 (1H, d, *J* = 7.7 Hz, H-1'').

Compound 20: Epigallocatechin

^{13}C NMR (151 MHz, CD_3OD-d_4) δ 78.6 (C-2), 66.2 (C-3), 27.8 (C-4), 156.7 (C-5), 95.0 (C-6), 156.3 (C-7), 94.5 (C-8), 156.0 (C-9), 98.7 (C-10), 130.2 (C-1'), 105.6 (C- 2', 6'), 145.3 (C-3', 5'), 132.3 (C-4'). ^{1}H-NMR (600 MHz, CD_3OD-d_4) δ 5.91 (1H, d, *J* = 2.3 Hz, H-8), 5.88 (1H, d, *J* = 2.3

Hz, H-6), 6.49 (2H, s, H-2', 6'), 4.73 (1H, m, H-2), 4.14 (1H, m, H-3).

Compound 21: Taxifolin-7-*O*-rhamnoside

^{13}C-NMR (101MHz, CD_3OD-d_4) δ 84.8 (C-2), 73.2 (C-3), 198.8 (C-4), 165.5 (C-5), 97.6 (C-6), 165.6 (C-7), 96.3 (C-8), 163.7 (C-9), 102.4 (C-10), 129.1 (C-1'), 115.3 (C-2'), 145.8 (C-3'), 146.7 (C-4'), 115.5 (C-5'), 120.3 (C-6'), 99.0 (C-1''), 71.1 (C-2''), 71.4 (C-3''), 73 (C-4''), 70.7 (C-5''), 17.5 (C-6''). ^{1}H-NMR (400MHz, CD_3OD-d_4) δ 6.19 (1H, d, *J* = 2.2 Hz, H-6), 6.15 (1H, d, *J* = 2.4 Hz, H-8), 4.59 (1H, s, H-3), 4.53 (1H, d, *J* = 11.6 Hz, H-2), 5.45 (1H, d, *J*= 5.4 Hz, H-1″), 1.20 (3H, d, *J*=6.3 Hz,H-6''), 3.94 – 3.92 (1H, m, H-2''), 3.74 (1H, dd, *J*=6.8, 2.9 Hz, H- 3''), 3.53 – 3.50 (1H, m, H- 5''), 3.42 – 3.24 (1H, m, H- 4'')

Compound 22: Quercetin 3-*O*-β-D-glucose-7-*O*-β-D-gentiobioside

^{13}C-NMR (101 MHz, CD_3OD-d_4) δ 157.4 (C-2), 134.1 (C-3), 178.1 (C-4), 161.3 (C-5), 99.5 (C-6), 163.3 (C-7), 94.9 (C-8), 156.5 (C-9), 106.1 (C-10), 121.7 (C-1'), 116.8 (C-2'), 145.0 (C-3'), 148.8 (C-4'), 115.5 (C-5'), 122.1 (C-6'), 104.0 (C-1''), 74.4 (C-2''), 77.9 (C-3''), 69.6 (C-4''), 77.4 (C-5''), 61.1 (C-6''), 100.8 (C-1'''), 73.5 (C-2'''), 76.5 (C-3'''), 69.0 (C-4'''), 75.8 (C-5'''), 68.4 (C-6'''), 101.4 (C-1''''), 73.8 (C-2''''), 76.8 (C-3''''), 70.4 (C-4''''), 77.0 (C-5''''), 61.4 (C-6''''). ^{1}H-NMR (400 MHz, CD_3OD-d_4) δ 7.62 (1H, d, *J* = 2.3 Hz, H-2'), 7.54 (1H, dd, *J* = 8.5, 2.3 Hz, H-6'), 6.83 (1H, d, *J* = 8.5 Hz, H-5'), 6.72 (1H, d, *J* = 2.1 Hz, H-8), 6.47 (1H, d, *J* = 2.1 Hz, H-6), 5.43 (1H, d, *J* = 7.5 Hz, H-1''), 5.04 (1H, d, *J* = 6.8 Hz, H-1'''), 4.15 (1H, d, *J* = 7.8 Hz, H-1''''),4·01– 2·98 (m, sugar-H).

Compound 23: Ampelopsin

^{13}C-NMR (101 MHz, CD_3OD-d_4) δ 84.7 (C-2), 73.1 (C-3), 197.8 (C-4), 164.7 (C-5), 96.7 (C-6), 168.2 (C-7), 95.7 (C-8), 163.8 (C-9), 101.2 (C-10), 128.5 (C-1'), 107.4 (C-2', 6'), 146.3 (C-3', 5'), 134.3 (C-4'). ^{1}H-NMR (400 MHz, CD_3OD-d_4) δ 6.64 (1H, dd, *J* = 13.2, 4.9 Hz, H-8), 5.38 (1H, s, H-6), 5.20 (1H, dd, *J* = 11.4, 4.7 Hz, H-2), 4.05 (1H, s, H-3).

Compound 24: Isorhamnetin

^{13}C-NMR (101 MHz, C_5D_5N-d_5) δ 150.5 (C-2), 138.0 (C-3), 177.4 (C-4), 162.5 (C-5), 99.3 (C-6), 165.7 (C-7), 94.5 (C-8), 157.5 (C-9), 104.5 (C-10), 123.1 (C-1'), 112.6 (C-2'), 147.6 (C-3'), 148.4 (C-4'), 116.7 (C-5'), 122.9 (C-6'), 56.0 (C-3'-OCH_3). ^{1}H NMR (400 MHz, CD_3OD-d_4) δ 7.99 (1H, d, *J* = 2.1 Hz, H-2'), 7.51 (1H, dd, *J* = 8.4Hz, 2.1Hz, H-6'), 6.99 (1H, d, *J* = 8.4Hz, H-5'), 6.47 (1H, d, *J* = 2.1 Hz, H-8), 6.15 (1H, d, *J* = 2.1 Hz, H-6), 3.15 (3H, s, OCH_3-3').

Compound 25: Taxifolin

^{13}C-NMR (101MHz, CD_3OD-d_4) δ 84.5 (C-2), 73.0 (C-3), 197.8 (C-4), 164.7 (C-5), 96.7 (C-6), 168.1 (C-7), 95.6 (C-8),163.9 (C-9), 99.9 (C-10), 129.2 (C-1'), 115.2 (C-2'), 145.7 (C-3'), 146.5 (C-4'), 115.4 (C-5'), 120.3 (C-6'). ^1H-NMR (400 MHz, CD_3OD-d_4) δ 6.92 (1H, s, H-2'), 6.81 (1H, d, J = 8.4 Hz, H-6'), 6.76 (1H, d, J = 8.4 Hz, H-5'), 5.88 (1H, d, J = 3.0 Hz, H-6), 5.84 (1H, d, J = 3.0 Hz, H-8), 4.59 (1H, s, H-3), 4.47 (1H, d, J = 11.6 Hz, H-2).
